# Early detection of hepatocellular carcinoma via liquid biopsy: panel of small extracellular vesicle‐derived long noncoding RNAs identified as markers

**DOI:** 10.1002/1878-0261.13049

**Published:** 2021-07-12

**Authors:** Soon Sun Kim, Geum Ok Baek, Ju A Son, Hye Ri Ahn, Moon Kyung Yoon, Hyo Jung Cho, Jung Hwan Yoon, Suk Woo Nam, Jae Youn Cheong, Jung Woo Eun

**Affiliations:** ^1^ Department of Gastroenterology Ajou University School of Medicine Suwon South Korea; ^2^ Department of Biomedical Sciences Ajou University Graduate School of Medicine Suwon South Korea; ^3^ Department of Pathology Functional RNomics Research Center College of Medicine The Catholic University of Korea Seoul South Korea; ^4^ Functional RNomics Research Center The Catholic University of Korea Seoul Korea; ^5^ Department of Biomedicine & Health Sciences Graduate School of Medicine The Catholic University of Korea Seoul Korea

**Keywords:** extracellular vesicle, hepatocellular carcinoma, liquid biopsy, lncRNA, MALAT1

## Abstract

This study investigated the diagnostic potential of serum small extracellular vesicle‐derived long noncoding RNAs (EV‐lncRNAs) for hepatocellular carcinoma (HCC). Driver oncogenic lncRNA candidates were selected by a comparative analysis of lncRNA expression profiles from two whole transcriptome human HCC datasets (Catholic_LIHC and TCGA_LIHC). Expression of selected lncRNAs in serum and small EVs was evaluated using quantitative reverse transcription PCR. Diagnostic power of serum EV‐lncRNAs for HCC was determined in the test (*n* = 44) and validation (*n* = 139) cohorts. Of the six promising driver onco‐lncRNAs, *DLEU2*, *HOTTIP*, *MALAT1,* and *SNHG1* exhibited favorable performance in the test cohort. In the validation cohort, serum EV‐*MALAT1* displayed excellent discriminant ability, while EV‐*DLEU2*, EV*‐HOTTIP,* and EV‐*SNHG1* showed good discriminant ability between HCC and non‐HCC. Furthermore, a panel combining EV‐*MALAT1* and EV‐*SNHG1* achieved the best area under the curve (AUC; 0.899, 95% CI = 0.816–0.982) for very early HCC, whereas a panel with EV‐*DLEU2* and alpha‐fetoprotein exhibited the best positivity (96%) in very early HCC. Serum small EV‐*MALAT1*, EV‐*DLEU2*, EV‐*HOTTIP,* and EV‐*SNHG1* may represent promising diagnostic markers for very early‐stage HCC.

## Graphical abstract

The present study investigates the diagnostic potential of serum small extracellular vesicle‐derived long noncoding RNAs (EV‐lncRNAs) for the early detection of hepatocellular carcinoma (HCC). We investigated driver lncRNA candidates by a comparative analysis of lncRNA expression profiles and identified that panels of four serum EV‐lncRNAs, EV‐*MALAT1*, EV‐*DLEU2*, EV‐*HOTTIP,* and EV‐*SNHG1,* with or without AFP, showed high positivity rates (86–96%) in very early‐stage HCC, thus highlighting their potential as promising diagnostic liquid biopsy markers.

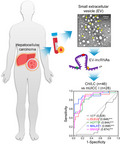

**Abbreviations**
AFP: alpha‐fetoprotein
AUC: area under the curve
avHCC: advanced hepatocellular carcinoma
Catholic_LIHC: the Catholic University of Korea's liver hepatocellular carcinoma project
CH: chronic hepatitis
CHB: chronic hepatitis B
CI: confidence interval
eHCC: early hepatocellular carcinoma
ER: endoplasmic reticulum
EV: extracellular vesicle
EV‐lncRNA: extracellular vesicle‐derived long noncoding RNA
GEO: gene expression omnibus
HCC: hepatocellular carcinoma
LC: liver cirrhosis
lncRNA: long noncoding RNA
mUICC: modified Union for International Cancer Control
NL: healthy control
NTA: nanoparticle tracking analysis
qRT-PCR: quantitative reverse transcription PCR
ROC: receiver operating characteristic
TCGA_LIHC: The Cancer Genome Atlas liver hepatocellular carcinoma project
TEM: transmission electron microscopy

## 1 Introduction

Primary liver cancer is one of the most commonly diagnosed cancers globally, with relatively high morbidity and mortality [1]. Hepatocellular carcinoma (HCC) is the most prevalent primary liver cancer accounting for approximately 75% of all liver cancer cases [2]. Despite surveillance with abdominal ultrasonography among the at‐risk populations, only 20% of HCC patients can afford the curative treatment through surgical resection, ablation treatment, or liver transplantation [3, 4]. Moreover, recently, the sensitivity and specificity of clinical screening and diagnostic techniques have been questioned, particularly for very early‐stage HCC. Hence, pressing need exists for the development of HCC diagnostic biomarkers with improved sensitivity and specificity to increase the opportunity for curative treatment and improve patient prognosis.

Tumor‐secreted extracellular vesicles (EVs) serve as critical intercellular communicator between tumor cells and stromal cells in local and distant tumor environments. EVs, detectable in all body fluids, are resistant to biological degradation and, thus, have been reported as promising biomarkers for monitoring cancer development, particularly in liquid biopsy approaches [5, 6].

Long noncoding RNAs (lncRNAs), a group of noncoding RNA more than 200 nucleotides in length, function as important players in chromatin modification, transcriptional inhibition, RNA processing, and gene activation by acting as decoys or signals [7, 8]. Recent studies indicated that HCC‐related lncRNAs such as *HOTAIR*, *HULC*, *H19,* and *MALAT1* have decisive regulatory roles in the development and progression of HCC, while their dysregulation is related to various biological processes, including differentiation, proliferation, apoptosis, invasion, and metastasis [9, 10]. Although lncRNAs have been primarily examined in the context of tissues, it is likely that they also exist in various body fluids, either free, bound to proteins, or coated by EVs, suggesting their usefulness as liquid‐based noninvasive markers for clinical use as a diagnostic and therapeutic target. However, the potential of dysregulated lncRNAs, particularly those in circulating tumor‐derived EVs, has not been fully explored to assess their potential as liquid biopsy biomarkers.

The current study, therefore, sought to investigate potential lncRNA biomarkers using HCC whole transcriptome sequencing datasets from The Cancer Genome Atlas liver hepatocellular carcinoma project (TCGA_LIHC) and the Catholic University of Korea's liver hepatocellular carcinoma project (Catholic_LIHC) and to subsequently validate their applicability as HCC diagnostic biomarkers. The significantly differently expressed lncRNAs between HCC tissues and nontumor liver tissues were analyzed, and six lncRNAs—*DLEU2*, *HOTTIP*, *MALAT1*, *NEAT1*, *SNHG1,* and *TUG1*—were identified as candidate biomarkers for HCC. Furthermore, serum small EV‐derived *DLEU2*, *HOTTIP*, *MALAT1,* and *SNHG1*, which showed good to excellent discriminating ability to diagnose very early HCC with or without alpha‐fetoprotein (AFP), were identified as potential liquid biopsy biomarkers. Overall, small EV‐derived lncRNAs could help diagnose very early‐stage HCC even in patients without AFP elevation.

## 2 Methods

### 2.1 LncRNA expression analyses

Next‐generation RNA‐sequencing datasets were acquired from the Catholic_LIHC and TCGA_LIHC. Public microarray datasets (GSE77314, GSE94660, and GSE124535) were obtained from the National Center for Biotechnology Information Gene Expression Omnibus (GEO). Two independent whole transcriptome data (Catholic_LIHC and TCGA_LIHC) were analyzed to identify overexpressed lncRNA candidates in HCC. Other datasets were then used to validate the expression of candidate lncRNAs. All dataset sequencing reads were quality‐controlled using fastqc (https://www.bioinformatics.babraham.ac.uk/projects/fastqc/), followed by mapping to human Gencode release version 22 using star software (https://code.google.com/archive/p/rna‐star/). The Lnc2Cancer 2.0 database was used to select the lncRNAs with the most significant functional roles in cancer. Hence, only lncRNAs registered in Lnc2Cancer2.0 were selected as final candidates.

### 2.2 Study population and definitions

Sera and the clinical information were collected from the Biobank of Ajou University Hospital, a member of Korea Biobank Network, between January 2014 and December 2018. The study population was divided into groups of healthy controls (NL), subjects with chronic hepatitis (CH), subjects with liver cirrhosis (LC), and subjects with HCC. Healthy controls were defined as 18‐ to 50‐year‐old subjects with no past medical history who attended regular medical check‐up in the Ajou Health Promotion Center and had normal health status. CH was diagnosed by hepatitis C virus RNA or serum hepatitis B surface antigen detection for more than six consecutive months, as well as elevated aminotransferase or alanine aminotransferase. LC was diagnosed based on clinical data, radiologic findings, and/or histological confirmations. HCC was diagnosed according to the American Association for the Study of Liver Diseases practice guideline [11]. Very early‐stage HCC was used to denote a single tumor of < 2 cm in diameter, equivalent to modified Union for International Cancer Control (mUICC) stage I. Overall survival was calculated based on time interval from HCC diagnosis to death from any cause. This study was approved by the Institutional Review Board of Ajou University Hospital, Suwon, South Korea (AJRIB‐BMR‐KSP‐18‐397 and AJIRB‐BMR‐KSP‐18‐299). Anonymous sera and clinical information were supplied by the Ajou Human Bio‐Resource Bank, and the requirement for informed consent was waived.

### 2.3 Cell culture

The human HCC cell lines (Huh‐7 and SNU449) were obtained from KCLB (Korean Cell Line Bank, Seoul, Korea). Immortalized normal hepatocytes (MIHA) were provided by Dr. Roy‐Chowdhury (Albert Einstein College of Medicine, Bronx, NY, USA). HCC and MIHA cells were maintained in Dulbecco's modified Eagle's medium (DMEM; GenDEPOT, Barker, TX, USA) or RPMI 1640 (GenDEPOT), supplemented with 10% fetal bovine serum (Invitrogen, Waltham, MA, USA) and 100 units·mL^−1^ penicillin–streptomycin (GenDEPOT) at 37 °C in a humidified incubator with 5% CO_2_.

### 2.4 Serum isolation and small EV extraction from patients

To compare lncRNA expression between serum and serum EV, blood samples were collected from patients, from which serum was isolated in 1.5 mL tubes and stored at −80 °C until use. Serum EV was extracted using ExoQuick (System Biosciences, Mountain View, CA, USA). The details of the modified protocol were described in our previous study [12].

### 2.5 Characterization of serum small EVs

The presence, and size, of small EVs was visualized by transmission electron microscopy (TEM) and nanoparticle tracking analysis (NTA). Next, protein markers for EVs and endoplasmic reticulum (ER) were detected and quantified in serum EVs and Huh‐7 cell lysate by western blotting. Briefly, cells were lysed with RIPA (radioimmunoprecipitation assay) buffer (100 µL; Thermo Scientific, Waltham, MA, USA) and sonicated briefly. Cell lysates were boiled in sample buffer, and 10 μg of each protein was subjected to sodium dodecyl sulfate/polyacrylamide gel electrophoresis. The protein concentration was measured by bicinchoninic acid protein assay (Thermo Scientific). Primary antibodies used in this study were rabbit anti‐CD9 (1:2000; ab92726; Abcam, Cambridge, UK), anti‐CD63 (1 : 1000, ab134045; Abcam), anti‐CD81 (1 : 250; 10630D; Invitrogen), and anti‐BiP/GRP78 (1 : 1000; 610979; BD Biosciences, San Jose, CA, USA).

### 2.6 RNA extraction and quantitative reverse transcription PCR

Serum RNA was extracted using the TRIzol‐LS reagent (Invitrogen), while RNA from small EVs was isolated from serum using the SeraMir™ Exosome RNA Amplification kit (System Biosciences), according to the manufacturer's instructions [12]. Next, serum RNA (500 ng) was reverse‐transcribed into cDNA using the PrimeScript™ RT Master mix (TaKaRa Bio, Otsu, Japan), whereas small EV‐derived RNA (50 ng) was reverse‐transcribed using the miScript II RT kit (QIAGEN, Hilden, Germany). cDNA was then used as templates for quantitative reverse transcription PCR (qRT‐PCR) with the amfiSure qGreen Q‐PCR Master Mix (GenDEPOT), which was performed on the ABI 7300 Real Time PCR System (Applied Biosystems™, Foster City, CA, USA). Primer information is summarized in Table S1. The $2^{‐\Delta \Delta C_{t}}$ method was used to determine the expression of each lncRNA relative to the internal control gene, *HMBS*. The detailed analysis methodology was described in a previous study [12].

### 2.7 EV uptake to recipient cells

The cell culture media were collected and centrifuged at 3000 ***g*** for 15 min to remove cells and cell debris. The supernatant was then mixed with the appropriate volume of ExoQuick and incubated at 4 °C overnight. The mixture was centrifuged at 1500 ***g*** for 30 min, and the supernatants were removed by aspiration. EV pellets were resuspended with 50 µL of PBS. The size of small EVs was visualized by NTA. To examine the uptake of EVs into recipient MIHA cells, MIHA cells were incubated with PBS or SNU449‐derived EVs (10 µg·mL^−1^) for 4 h at 37 °C. The cells were then washed with PBS, fixed in 4% paraformaldehyde, permeabilized in 0.25% Triton X‐100, and incubated with primary and secondary antibodies. Primary antibodies were anti‐CD81 (1 : 20, MA5‐13548; Invitrogen) and anti‐EEA1 (1 : 50, #3288, Cell Signaling Technology, Danvers, MA, USA). Secondary antibodies were Alexa Fluor 488 donkey anti‐mouse IgG and Alexa Fluor Texas Red donkey anti‐rabbit IgG (Invitrogen). EV uptake was observed by fluorescence microscopy (IX71; Olympus, Tokyo, Japan). To inhibit endocytosis of EVs, MIHA cells were pretreated with 30 μm pitstop 2 (Sigma, St. Louis, MO, USA) or DMSO (0.1%) for 30 min at 37 °C prior to the addition of SNU449‐EVs. After incubation, total RNA from the cells was isolated followed by cDNA synthesis. qRT‐PCR was performed to investigate the expression levels of lncRNAs as described above.

### 2.8 Statistical analysis

All statistical analyses were conducted with ibm spss software version 22.0 (SPSS Inc., Chicago, IL, USA) and graphpad prism version 7.01 (GraphPad Software, San Diego, CA, USA). *P* value < 0.05 was considered statistically significant. A chi‐square test (two‐sided) was performed for intergroup comparisons of categorical parameters, independent sample *t*‐test or Welch's *t*‐test was applied for continuous variables. ROC curves were constructed to define area under the curves (AUCs) with 95% confidence interval (CI) in each comparison group. The Kaplan–Meier survival curves with the log‐rank test were performed to assess significant prognostic power between two patient groups.

## 3 Results

### 3.1 Identification of candidate lncRNAs for HCC using integrative analysis of tissue‐based RNA‐sequencing datasets

**Fig. 1:**
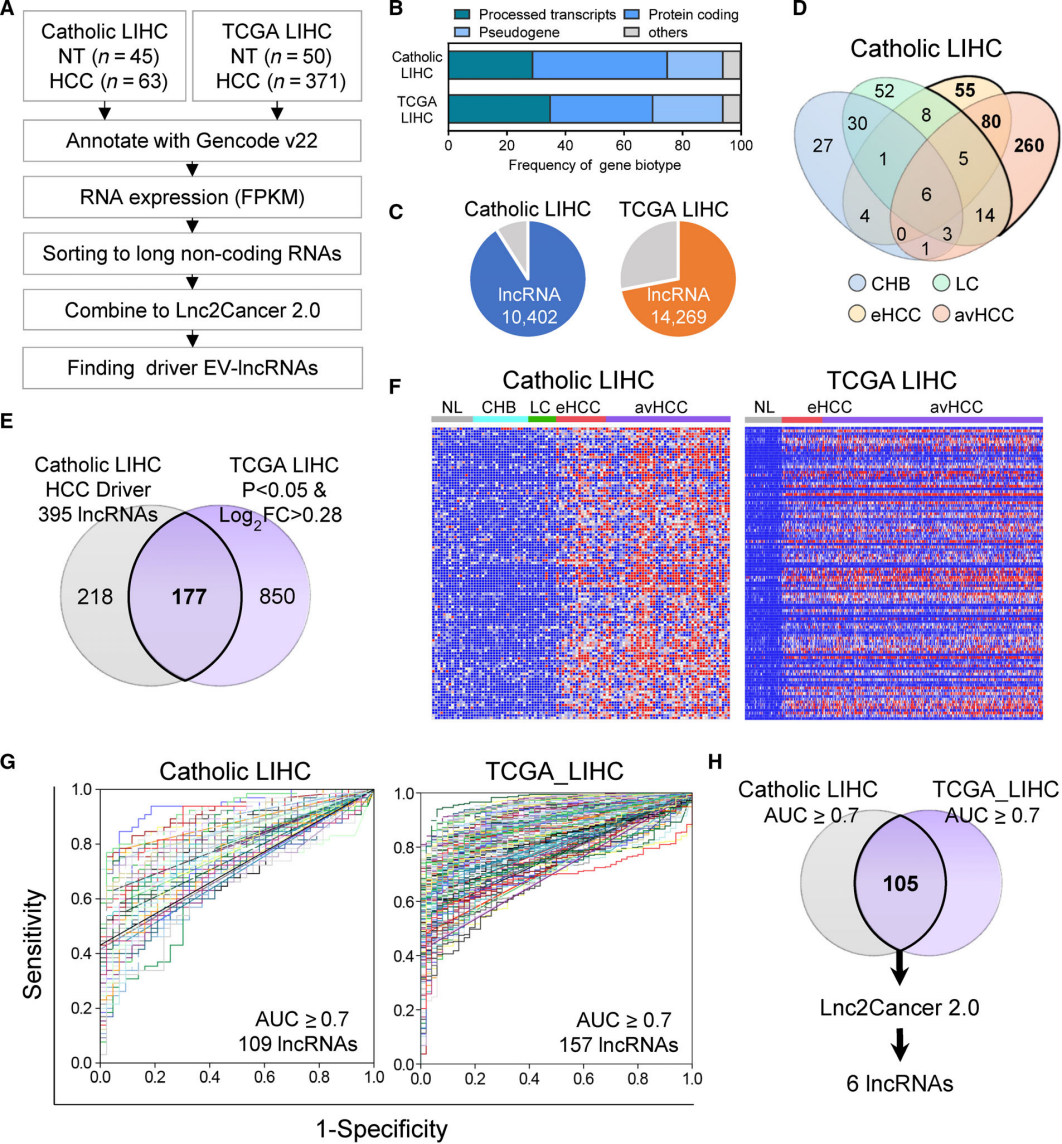
Integrative analysis of tissue‐based RNA‐sequencing datasets from two independent cohorts to identify novel serum EV‐derived lncRNAs of HCC. (A) Strategy to identify novel serum EV‐derived lncRNAs for diagnosing HCC. (B) The stacked bar chart shows the frequency of gene biotype in Catholic_LIHC and TCGA_LIHC datasets. (C) Pie chart distribution for lncRNAs identified in Catholic_LIHC and TCGA_LIHC datasets, respectively. (D) Venn diagram analysis of significantly overexpressed lncRNAs in each disease status compared with normal subjects in the Catholic_LIHC cohort. (E) Venn diagram showing overlapping outlier lncRNAs between two different RNA‐Seq datasets (Catholic_LIHC and TCGA_LIHC). (F) Heatmaps of 177 HCC‐associated lncRNA signatures in Catholic_LIHC and TCGA_LIHC datasets (G) Receiver operating curves of 177 common lncRNAs with AUC ≥ 0.7 in the two RNA‐sequencing datasets. (H) Selection of lncRNAs (6) registered in Lnc2Cancer from 105 lncRNAs with AUC ≥ 0.7 in both datasets.

Analysis of the Catholic_LIHC and TCGA_LIHC RNA‐sequencing datasets (Fig. 1A) identified 10 402 and 14 269 lncRNAs, respectively, by gene biotype (Fig. 1B,C). Subsequently, the Catholic_LIHC was divided into chronic hepatitis B (CHB), LC, early HCC (eHCC), and advanced HCC (avHCC), with 395 lncRNAs identified as expressed only in eHCC or avHCC (Fig. 1D). From the TCGA_LIHC dataset, 1027 lncRNAs were significantly differentially expressed between the HCC and nontumor samples (*P* < 0.05; Log2 fold change > 0.28, Fig. 1E). Of these lncRNAs, the heatmaps for 177 common lncRNAs clearly distinguished between the HCC and non‐HCC specimens (Fig. 1F). With ROC analysis for differentiation of HCC, we identified 105 lncRNAs with AUC ≥ 0.7 in both Catholic_LIHC and TCGA_LIHC datasets (Fig. 1G). Finally, six lncRNAs (*DLEU2*, *HOTTIP*, *MALAT1*, *NEAT1*, *SNHG1,* and *TUG1*), registered in Lnc2Cancer 2.0, a database offering comprehensive experimentally supported associations between lncRNA and human cancer, were selected as candidate lncRNAs for the diagnosis of HCC (Fig. 1H).

**Fig. 2:**
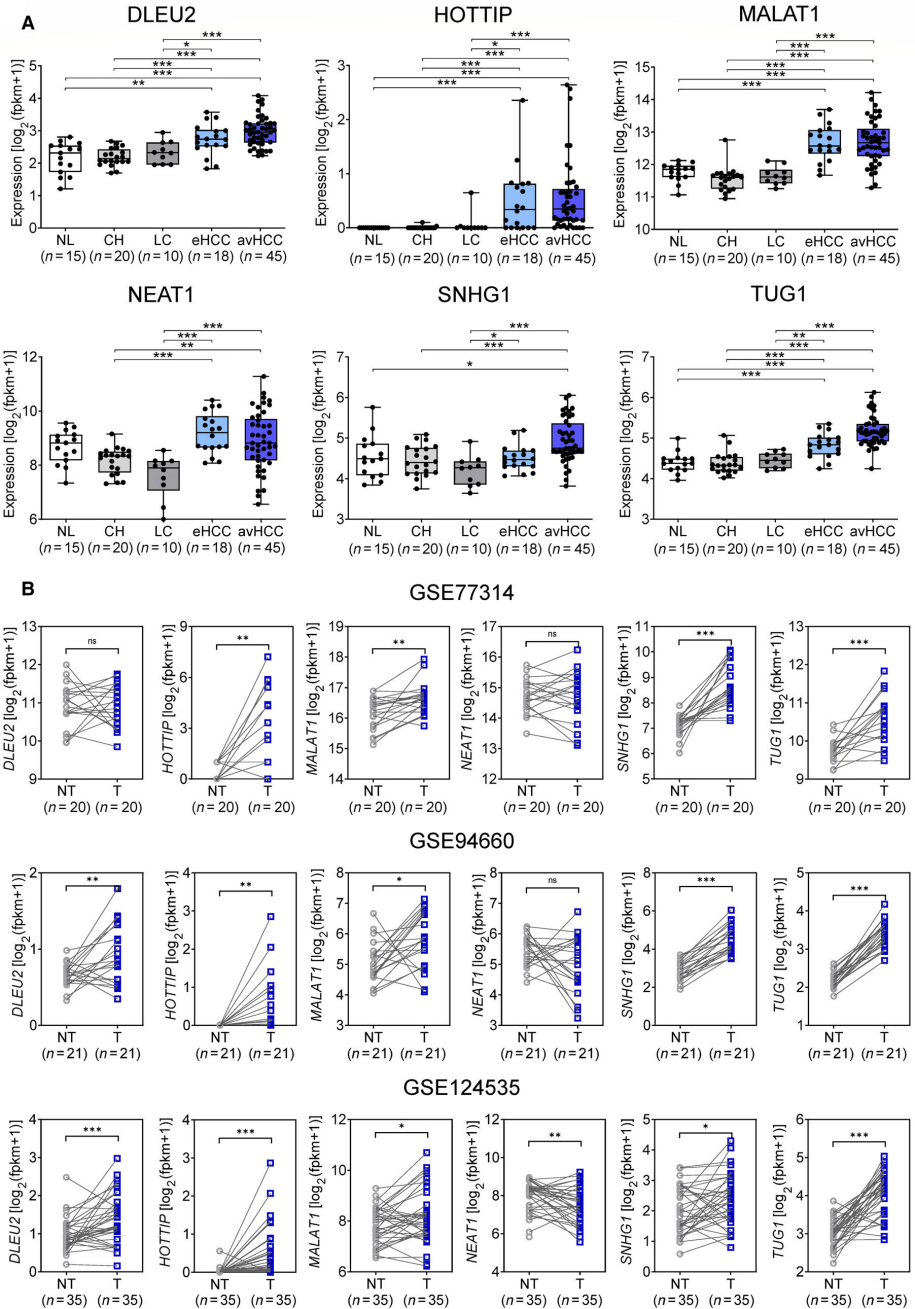
Differential gene expression of six lncRNA candidates in HCC (A) according to liver disease status in Catholic_LIHC cohort and (B) in the three independent RNA‐seq datasets. (Welch's *t*‐test; *P < 0.05, **P < 0.01, and ***P < 0.001).

Next, we analyzed the gene expression of these six candidate lncRNAs based on liver disease status in the Catholic_LIHC dataset. All six lncRNAs were differentially expressed in the five stages of liver disease (Fig. 2A). In addition, the expression of these six candidate lncRNAs in HCC and nontumor tissues in other GEO RNA‐sequencing datasets (GSE77314, GSE94660, and GSE124535) confirmed the higher expression of most candidate lncRNAs in tumor tissues, save for *DLEU2* and *NEAT1* in GSE77314 and *NEAT1* in GSE94660 (Fig. 2B).

### 3.2 Measurement of candidate lncRNA expression in serum and serum‐derived EVs in the test cohort

**Fig. 3:**
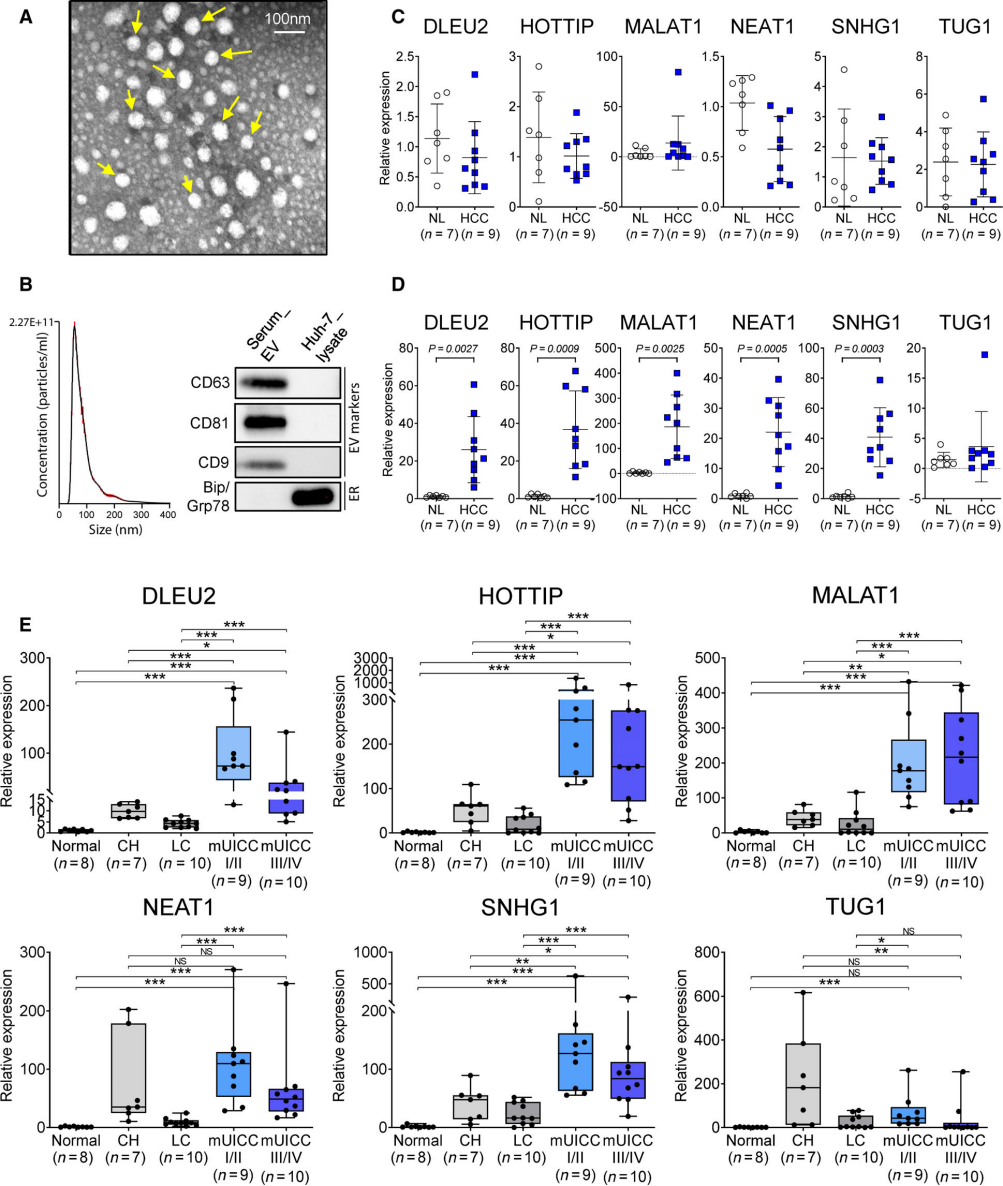
Expression of the six lncRNAs in sera and serum‐derived extracellular vesicles (EVs). (A) Transmission electron microscopy analysis showing EV morphology in the serum of HCC patients (scale bar indicated 100 nm). (B) Representative NTA and immunoblots of EV and ER markers from serum EV and Huh‐7 cell lysate. (C) Scatter plots for the expression of six lncRNAs in the (C) serum and (D) serum‐derived EVs of healthy controls (*n* = 7) and subjects with HCC (*n* = 9), and (E) differential gene expression of six serum EV‐lncRNAs according to liver disease status in the test cohort (*n* = 44). Black horizontal lines indicate sample means. Target gene expression was calculated relative to that of HMBS (Welch's *t*‐test; *P < 0.05, **P < 0.01, and ***P < 0.001).

To confirm the usefulness of the candidate lncRNAs as liquid biopsy biomarkers for HCC, we quantified their concentration in the serum and serum‐derived EVs of seven NL and nine HCC subjects. EV characterization was confirmed by TEM (Fig. 3A), NTA, and immunoblotting for EV markers and ER markers (Fig. 3B and Fig. S1A). qRT‐PCR analysis revealed no significant differences in the level of serum‐derived lncRNAs of the two groups (Fig. 3C), whereas the levels of serum EV‐derived lncRNAs (except for *TUG1*) were significantly higher in subjects with HCC than in NL (Fig. 3D). Further evaluation of the six serum EV‐derived lncRNAs in the test cohort (*n* = 44) consisting of NL (*n* = 8), CH (*n* = 7), LC (*n* = 10), mUICC I/II HCC (*n* = 9), and mUICC III/IV HCC (*n* = 10) identified four lncRNAs—serum EV‐derived *DLEU2* (EV‐*DLEU2*), *HOTTIP* (EV‐*HOTTIP*), *MALAT1* (EV‐*MALAT1*), and *SNHG1* (EV‐*SNHG1*)—as significantly discriminatory for the HCC and non‐HCC samples.

To confirm that the four lncRNAs originated from HCC, we investigated whether normal liver cells are capable of taking up HCC‐derived EVs. First, we compared four lncRNA expression in MIHA‐EV and SNU449‐EV. All lncRNA expressions in SNU449‐EV were higher than in MIHA‐EV (Fig. S1B). After SNU449‐EV was treated to MIHA, resulting images clearly showed overlapping of green (CD8; EV marker), red (EEA1; endosome marker), and blue (DAPI) fluorescence indicating successful delivery of EV to MIHA (Fig. S1C). Moreover, the relative expression levels of *DLEU2*, *HOTTIP*, *MALAT1,* and *SNHG1* in EV‐treated MIHA were significantly increased compared to those in PBS‐treated MIHA, but also significantly decreased by endocytosis inhibitor (Fig. S1D). These results suggest that the four candidate lncRNAs originated from EVs and may participate in crosstalk between HCC and normal liver cells. Hence, these were the four candidates ultimately selected for further validation (Fig. 3E).

### 3.3 Validation of the four serum EV‐lncRNAs for HCC diagnosis

**Fig. 4:**
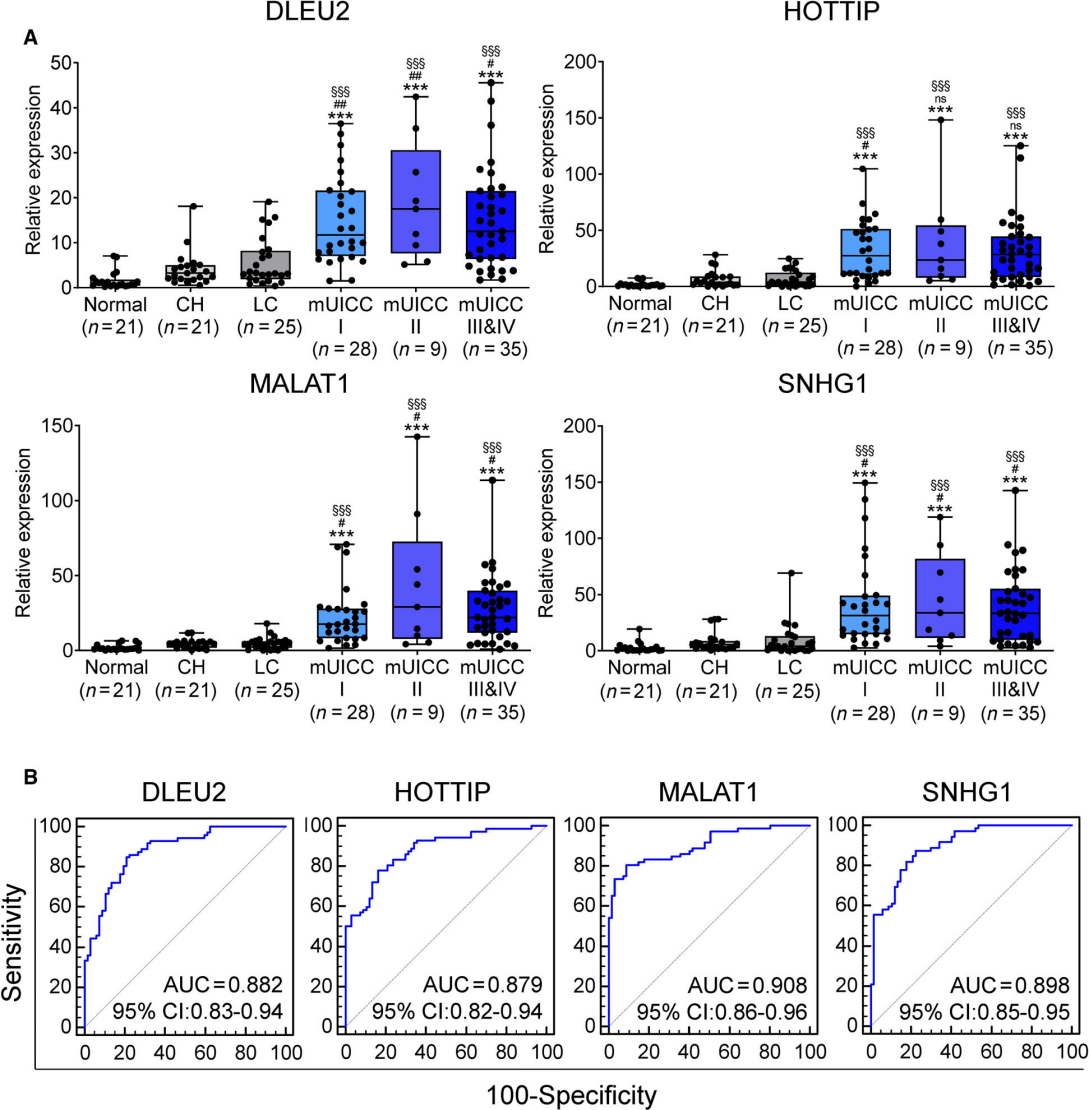
Diagnostic performance of the final four serum EV‐lncRNAs for HCC in the validation cohort. (A) Box plot showing the expression of four serum EV‐lncRNAs according to liver disease status in the validation cohort. Black horizontal lines indicate means, while error bars represent standard error of the mean (Welch's *t*‐test; compared to NL, ***P < 0.001; compared to CH, ^#^ *P* < 0.05, ^##^ *P* < 0.01; compared to LC; ^§§§^ *P* < 0.001). (B) ROC curves for the four serum EV‐lncRNAs in the validation cohort.

We then analyzed the diagnostic performance of EV‐*DLEU2*, EV‐*HOTTIP*, EV‐*MALAT1*, and EV‐*SNHG1* for HCC in the validation cohort, comprising 72 subjects with HCC and 67 subjects without HCC. Table 1 summarizes the clinical baseline characteristics of the validation cohort. The proportions of subjects with mUICC stage I, II, III, IVA, and IVB HCC were 39%, 13%, 28%, 14%, and 7%, respectively. The expression levels of the four EV‐derived lncRNAs were significantly higher in patients with all mUICC stages compared with NL, CH, and LC subjects (Fig. 4A). Meanwhile, their expression levels did not differ significantly between the mUICC and BCLC stages or based on vascular invasion (Fig. 4A and Fig. S2).

**Table 1:** Baseline characteristics of patients selected for the validation cohort. CH, chronic hepatitis; AST, aspartate transaminase; ALT, alanine transaminase; HBV, hepatitis B virus; HCV, hepatitis C virus; INR, international normalized ratio; RFA, radiofrequency ablation; TACE, transarterial chemoembolization; BSC, best supportive care.

Variables	Validation cohort (*n* = 139)			
	NL (*n* = 21)	CH (*n* = 21)	LC (*n* = 25)	HCC (*n* = 72)
Age (years), mean ± SD	34.0 ± 7.3	49.8 ± 1.3	48.3 ± 7.7	54.8 ± 8.9
Male sex, *n* (%)	2 (9.52)	12 (57.14)	17 (68.00)	58 (80.5)
AST, IU·mL^−1^	17.1 ± 4.1	75.3 ± 53.0	51.8 ± 22.6	73.9 ± 93.5
ALT, IU·mL^−1^	13.9 ± 8.4	118.8 ± 128.6	71.5 ± 49.7	48.9 ± 63.5
Platelet, ×10^9^ L^−1^	284.8 ± 43.5	187.7 ± 51.4	133.9 ± 71.8	166.3 ± 83.9
AFP (ng·mL^−1^), mean ± SD	1.6 ± 0.6	21.8 ± 26.9	85.5 ± 148.4	3539.7 ± 13 921.6
Etiology, *n*				
HBV				64 (88.9)
HCV				4 (5.5)
Alcohol				3 (4.2)
Others				1 (1.4)
Albumin (g·L^−1^), mean ± SD				4.29 ± 0.58
Bilirubin (mg·dL^−1^), mean ± SD				1.58 ± 3.96
INR, mean ± SD				1.18 ± 0.19
BCLC stage, *n* (%)				
0				26 (36.1)
A				9 (12.5)
B				9 (12.5)
C				27 (37.5)
D				1 (1.4)
Modified UICC stage, *n* (%)				
I				28 (38.9)
II				9 (12.5)
III				20 (27.8)
IVA				10 (13.9)
IVB				5 (6.9)
Vascular invasion, *n* (%)				22 (30.6)
HCC treatment, *n* (%)				
Resection				43 (59.7)
Liver transplantation				4 (5.6)
RFA				3 (4.2)
TACE				4 (5.6)
RFA + TACE				2 (2.8)
TACE + Resection				1 (1.4)
Sorafenib				8 (11.1)
BSC or unknown				7 (9.7)

ROC curve analysis further showed the high discriminatory abilities of the four EV‐derived lncRNAs for the diagnosis of HCC, with EV‐*MALAT1* emerging as the best (AUC = 0.908, 95% CI = 0.86–0.96; Fig. 4B). With the optimal cutoff values—5.5318‐fold for EV‐*DLEU2,* 9.2165‐fold for EV‐*HOTTIP*, 7.3752‐fold for EV‐*MALAT1,* and 7.7427‐fold for EV‐*SNHG1*—EV‐*MALAT1* also exhibited the highest sensitivity (92.1%) and negative predictive value (92.5%), while EV‐*SNHG1* showed the best specificity (85.2%) and positive predictive value (87.5%; Table 2).

**Table 2:** Comparative analysis between diagnosing HCC using serum EV markers and AFP. PPV, positive predictive value; NPV, negative predictive value.

	*P* vs. AFP	AUC	95% CI	Sensitivity (%)	Specificity (%)	PPV (%)	NPV (%)
HCC vs. NL/CH/LC							
AFP (20 ng·mL^−1^)	1.0000	0.647	0.549–0.738	48.936	46.739	31.944	64.179
DLEU2 (5.5318‐fold)	< 0.0001	0.882	0.827–0.937	80.263	82.54	84.722	77.612
HOTTIP (9.2165‐fold)	< 0.0001	0.879	0.823–0.935	82.253	77.465	79.856	48.921
MALAT1 (7.3752‐fold)	< 0.0001	0.908	0.860–0.956	92.063	81.579	80.556	92.537
SNHG1 (7.7427‐fold)	< 0.0001	0.898	0.848–0.948	80.769	85.246	87.500	77.612
HCC vs. CH/LC							
AFP (20 ng·mL^−1^)	1.0000	0.506	0.391–0.611	48.936	46.739	31.944	64.179
DLEU2 (5.5318‐fold)	< 0.0001	0.844	0.773–0.916	82.432	83.077	84.722	80.597
HOTTIP (9.2165‐fold)	< 0.0001	0.844	0.776–0.913	82.090	77.465	77.465	82.090
MALAT1 (7.3752‐fold)	< 0.0001	0.886	0.827–0.945	92.063	81.579	80.556	92.537
SNHG1 (7.7427‐fold)	< 0.0001	0.868	0.804–0.933	82.895	85.714	87.500	80.597
mUICC I/II vs. NL/CH/LC							
AFP (20 ng·mL^−1^)	1.0000	0.531	0.413–0.638	11.111	55.844	8.108	64.179
DLEU2 (5.5318‐fold)	< 0.0001	0.896	0.833–0.958	69.388	94.545	91.892	77.612
HOTTIP (9.2165‐fold)	< 0.0001	0.882	0.813–0.952	71.429	88.710	81.081	82.090
MALAT1 (7.3752‐fold)	< 0.0001	0.917	0.858–0.975	85.714	89.855	81.081	92.537
SNHG1 (7.7427‐fold)	< 0.0001	0.902	0.845–0.959	68.750	92.857	89.189	77.612
mUICC I/II vs. CH/LC							
AFP (20 ng·mL^−1^)	1	0.351	0.229–0.473	11.111	39.286	8.108	47.826
DLEU2 (5.5318‐fold)	0.0032	0.862	0.781–0.943	72.340	91.667	91.892	71.739
HOTTIP (9.2165‐fold)	0.0073	0.848	0.764–0.931	71.429	82.927	81.081	73.913
MALAT1 (7.3752‐fold)	0.0016	0.894	0.820–0.967	85.714	85.417	81.081	89.130
SNHG1 (7.7427‐fold)	0.0035	0.873	0.798–0.947	71.739	89.189	89.189	71.739
mUICC I vs. NL/CH/LC							
AFP (20 ng·mL^−1^)	1.0000	0.508	0.384–0.620	7.692	62.319	7.143	64.179
DLEU2 (5.5318‐fold)	< 0.0001	0.885	0.812–0.958	63.415	96.296	92.857	77.612
HOTTIP (9.2165‐fold)	< 0.0001	0.878	0.796–0.960	65.714	91.667	82.143	82.090
MALAT1 (7.3752‐fold)	< 0.0001	0.920	0.854–0.986	82.143	92.537	82.143	92.537
SNHG1 (7.7427‐fold)	< 0.0001	0.904	0.842–0.965	62.500	94.545	81.053	42.105
mUICC I vs. CH/LC							
AFP (20 ng·mL^−1^)	1.0000	0.328	0.205–0.452	7.692	45.833	7.143	47.826
DLEU2 (5.5318‐fold)	0.0181	0.848	0.753–0.943	66.667	94.286	92.857	71.739
HOTTIP (9.2165‐fold)	0.0237	0.844	0.748–0.940	65.714	87.179	82.143	79.913
MALAT1 (7.3752‐fold)	0.0050	0.898	0.816–0.981	82.143	89.130	82.143	89.130
SNHG1 (7.7427‐fold)	0.0089	0.874	0.795–0.954	65.789	91.667	89.286	71.739

Considering the uneven age distribution between the groups, we also assessed whether EV‐lncRNA expression varied depending on patient age. However, results show that EV‐lncRNA levels were comparable between different age groups, thereby confirming that the observed differences in EV‐lncRNA levels were based on HCC (Fig. S3).

### 3.4 Performance of EV‐*DLEU2*, EV‐*HOTTIP*, EV‐*MALAT1,* and EV‐*SNHG1* in the diagnosis of very early HCC

**Fig. 5:**
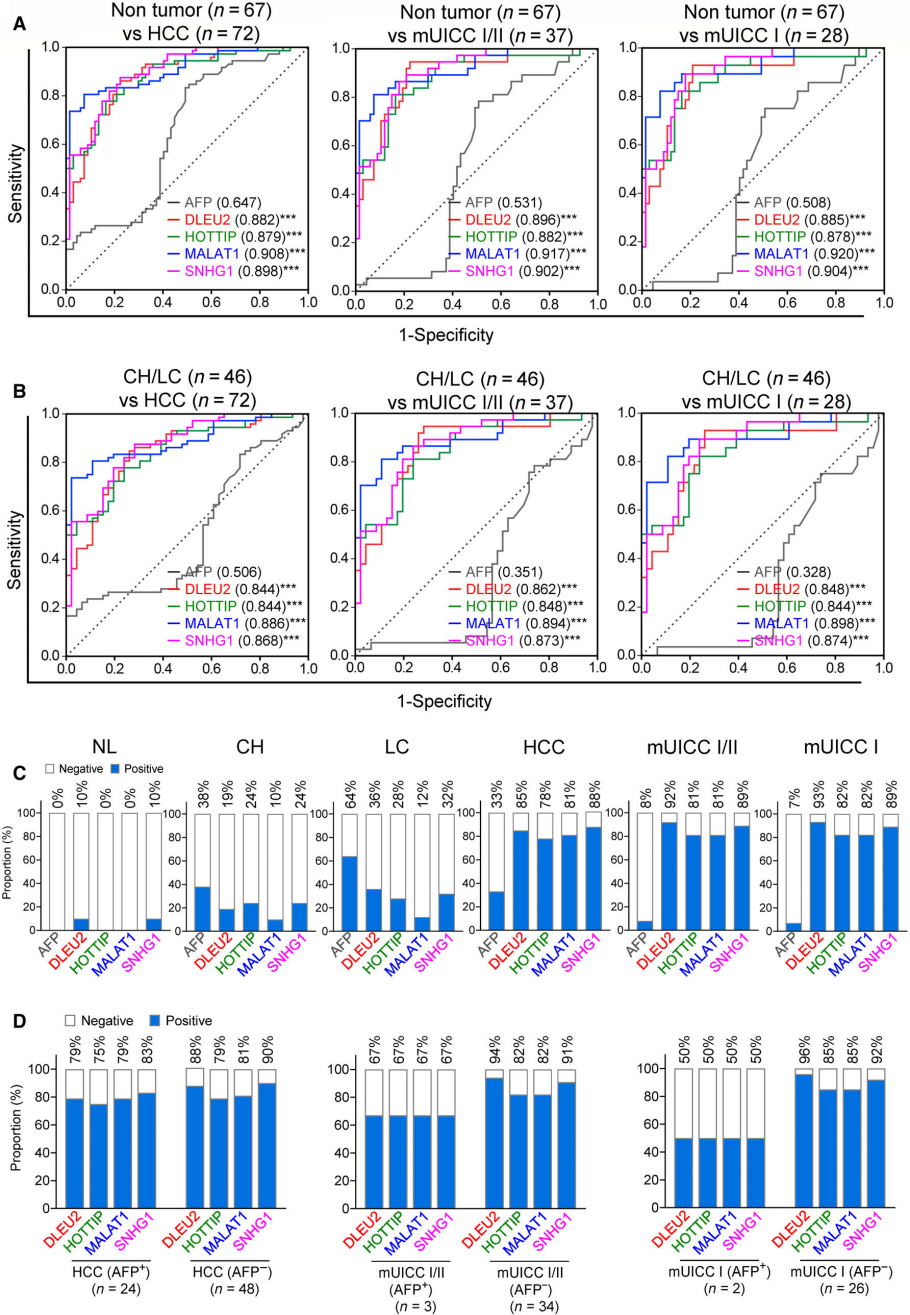
Diagnostic outcomes of four serum EV‐lncRNAs for all‐stage HCC and very early‐stage HCC. AUCs for diagnosing HCC at all stages (left panel), HCC with mUICC stage I or II (middle panel), and HCC with mUICC stage I (right panel) from (A) nontumor subjects, and (B) patients at high risk of developing HCC (CH and LC) (two‐tailed *t*‐test; compared to AUC of AFP, ***P < 0.001). Bar chart showing a positive rate for AFP, *DLEU2*, *HOTTIP*, *MALAT1,* and *SNHG1* in (C) patients with various liver disease status, and (D) patients with HCC, mUICC I/II, and mUICC I. The cutoff for positivity was defined as 5.5318‐fold for EV‐*DLEU2*, 9.2165‐fold for EV‐*HOTTIP*, 7.3752‐fold for EV‐*MALAT1*, 7.7427‐fold for EV‐*SNHG1,* and 20 ng·mL^−1^ for AFP level.

To assess EV‐*DLEU2*, EV‐*HOTTIP*, EV‐*MALAT1*, and EV‐*SNHG1* as diagnostic biomarkers for very early HCC, we compared the diagnostic abilities of EV‐derived lncRNAs for mUICC stage I, with that of AFP. EV‐*DLEU2*, EV‐*HOTTIP*, EV‐*MALAT1,* and EV‐*SNHG1* had significantly better performance than AFP for detection of very early HCC (Fig. 5A,B). Specifically, AUCs for mUICC stage I HCC (vs. NL, CH, and LC) were 0.508, 0.885, 0.878, 0.92, and 0.904 for AFP, EV‐*DLEU2*, EV‐*HOTTIP*, EV‐*MALAT1,* and EV‐*SNHG1*, respectively (Table 2). Additionally, EV‐derived lncRNAs showed a higher negative rate in CH and LC groups and a higher positive rate in the HCC group than AFP (Fig. 5C). Furthermore, EV‐*DLEU2*, EV‐*HOTTIP*, EV‐*MALAT1,* and EV‐*SNHG1* showed comparable positivity in AFP‐positive HCC (> 20 ng·mL^−1^) as well as AFP‐negative HCC (≤ 20 ng·mL^−1^) with the optimal cutoff values—5.5318‐fold for EV‐*DLEU2*, 9.2165‐fold for EV‐*HOTTIP*, 7.3752‐fold for EV‐*MALAT1,* and 7.7427‐fold for EV‐*SNHG1*. In the subgroup analysis of mUICC stage I or II, EV‐derived lncRNAs had a higher positive rate in AFP‐negative HCC than in AFP‐positive HCC (Fig. 5D).

### 3.5 Performance of biomarker panels comprising different combinations of serum AFP, EV‐*DLEU2*, EV‐*HOTTIP*, EV‐*MALAT1,* and EV‐*SNHG1* for the diagnosis of HCC and very early HCC

**Fig. 6:**
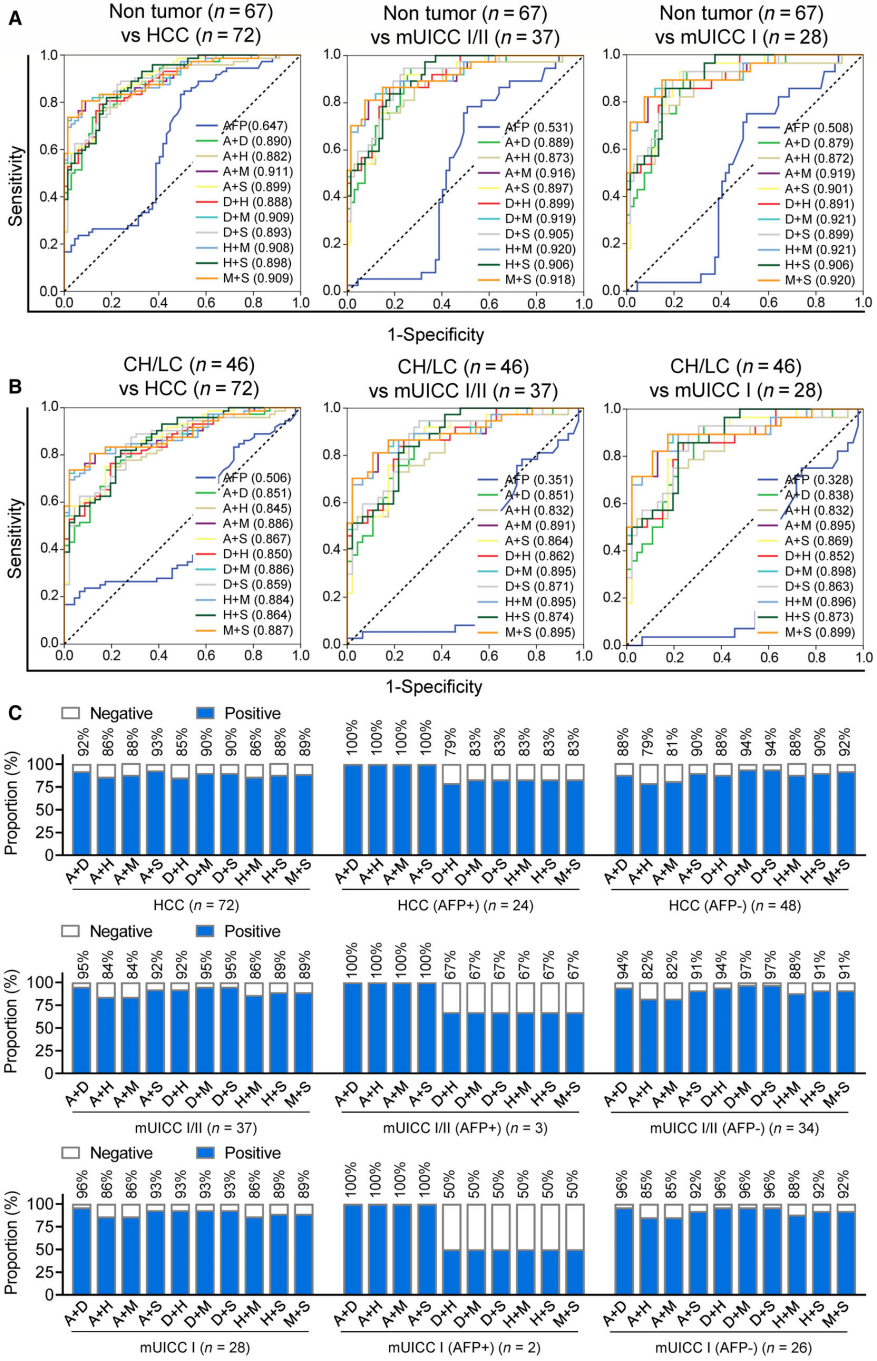
Combinations of AFP with four serum EV‐lncRNAs for the diagnosis of HCC and early‐stage HCC in the validation cohort. AUCs for the combination of two markers for diagnosing all‐stage HCC (left), HCC with mUICC stage I or II (middle), and HCC with mUICC stage I (right) versus (A) all controls (normal, chronic hepatitis, and liver cirrhosis), and (B) patients at high risk of developing HCC (chronic hepatitis and liver cirrhosis) (two‐tailed *t*‐test; compared to AUC of AFP, all comparison values were determined as *P* < 0.0001). (C) Bar chart showing a positive rate for the combination of two markers by AFP status in subjects with all‐stage HCC (top), mUICC I/II (middle), and mUICC I (bottom).

Next, we examined several combinations of AFP and EV‐lncRNAs to identify the optimal biomarker panel for diagnosing HCC (Fig. 6A,B; Table S2). In terms of all‐stage HCC diagnosis, the combination of AFP and EV‐*MALAT1* showed the best AUC (0.911, 95% CI 0.864–0.958) in a model of HCC vs. nontumor, while the EV‐*MALAT1* and EV‐*SNHG1* combination showed the best AUC (0.887, 95% CI 0.828–0.945) in a model of HCC vs. CH/LC. Moreover, for very early HCC (mUICC stage I), the combinations of EV‐*DLEU2* and EV‐*MALAT1*, and EV‐*HOTTIP* and EV‐*MALAT1* exhibited the best AUCs (0.921, 95% CI 0.854–0.987, and 0.921, 95% CI 0.856–0.986, respectively) in a model of HCC vs. nontumor, whereas the combination of EV‐*MALAT1* and EV‐*SNHG1* had the best AUC (0.899, 95% CI 0.816–0.982) in a model of HCC vs. CH/LC.

Still further, the AFP and EV‐*DLEU2* combination exhibited the highest positivity for all‐stage (92%) and mUICC stage I HCC (96%), whereas EV‐*DLEU2* and AFP or EV*‐HOTTIP* or EV‐*SNHG1* showed the highest positivity (95% for each combination) for mUICC stage I or II HCC. In fact, even in very early HCCs with low AFP levels (≤ 20 ng·mL^−1^), panels with EV‐lncRNAs showed high positivity (88–96%; Fig. 6C).

### 3.6 Prognostic implication of EV‐lncRNA expression

**Fig. 7:**
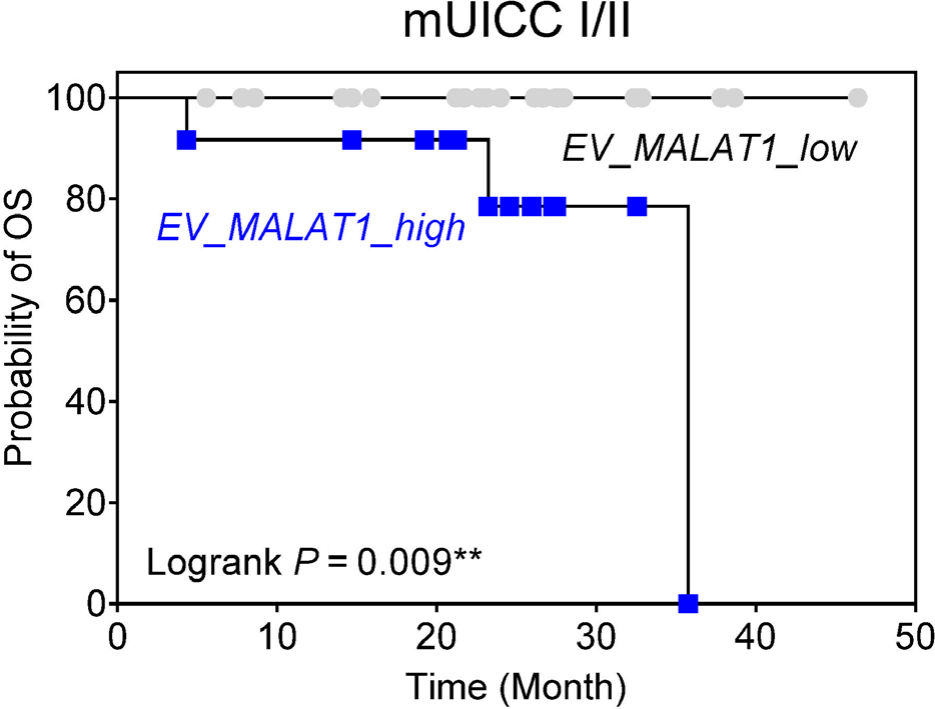
Prognostic implication of EV‐*MALAT1* expression. Kaplan–Meier survival curve of overall survival based on EV‐*MALAT1* level in subjects with mUICC I/II HCC in the validation cohort. ‘High’ and ‘low’ expression was defined based on the median expression level of EV‐*MALAT1* (27.732‐fold) in patients with HCC.

Finally, we examined the prognostic impact of EV‐lncRNA in the validation cohort. None of the candidate EV‐lncRNAs were significantly related to any of the 72 HCC patients' overall survival (Fig. S4). Meanwhile, in patients with mUICC stage I/II HCC, the high expression of EV‐*MALAT1* (≥ 27.732‐fold) was significantly associated with poor overall survival (log‐rank *P* = 0.009; Fig. 7). However, in patients with mUICC stage III/IV HCC, no single EV‐lncRNA was related to overall survival.

## 4 Discussion

This study compared the lncRNA expression between HCC and non‐HCC tissues using two RNA‐sequencing datasets, resulting in the identification of six lncRNAs (*DLEU2*, *HOTTIP*, *MALAT1*, *NEAT1, SNHG1,* and *TUG1)* as candidate biomarkers for HCC diagnosis. Among them, *DLEU2*, *HOTTIP*, *MALAT1,* and *SNHG1* were significantly elevated in small EV of serum from patients with HCC compared to those without HCC. Serum small EV‐derived *MALAT1* displayed excellent discriminant capacity, whereas small EV‐derived *DLEU2*, *HOTTIP,* and *SNHG1* showed good discriminant ability between HCC and non‐HCC. Additionally, a combination panel of EV‐*MALAT1* and EV‐*SNHG1* achieved the best AUC for very early HCC. In fact, even in very early HCC with low AFP levels (≤ 20 ng·mL^−1^), panels combining EV‐lncRNAs showed high positivity (88–96%), suggesting the utility of EV‐lncRNAs as diagnostic liquid biopsy biomarkers for AFP‐negative HCC.

*MALAT1* is a representative onco‐lncRNA that is upregulated in many solid carcinomas [13]. It stimulates tumor growth, metastasis, and chromosomal instability through multiple mechanisms in different tissues, including modulating alternative splicing of oncogenic mRNAs, attaching to active regions of the chromosome, and recruiting serine/arginine‐rich family proteins [14, 15, 16]. Although several studies have implicated it in the prognosis and development of HCC [17, 18, 19], few studies have actually reported the diagnostic value of circulating *MALAT1* for HCC diagnosis. A recent study evaluating eight serum lncRNAs including serum *MALAT1* reported an acceptable AUC (0.733, 95% CI = 0.676–0.790) with 59.7% sensitivity and 75.7% specificity in a model of HCC vs. LC, CHB, and healthy controls; however, this was lower than the AUC for AFP (0.811) [20]. Similarly, another study measuring plasma *MALAT1* reported an AUC of 0.66 with 51.1% sensitivity and 89.3% specificity, which was also lower than that of AFP (sensitivity 73.3%, specificity 75%, and AUC 0.7) [21]. Meanwhile, Yuan *et al*. [22] quantified the level of 10 lncRNAs including *MALAT1*, in the plasma of 100 subjects with HCC, 100 subjects with CH, and 100 healthy controls, and reported no significant difference in plasma *MALAT1* levels among the three groups. Therefore, to the best of our knowledge, the current study is the first to report the excellent diagnostic value of EV‐derived *MALAT1* in HCC with an AUC of 0.908, sensitivity of 92.1%, and specificity of 81.6% in an HCC vs. NL/CH/LC model.

*DLEU2* was also overexpressed in HCC tissues, particularly in large tumors with vascular invasion, and advanced‐stage [23]. Recently, HBx was reported to bind the promoter region of the *DLEU2* lncRNA, thereby enhancing *DLEU2* transcription and inducing the accumulation of *DLEU2* RNA. Moreover, the interaction of *DLEU2* with HBx and the enhancer of zeste homolog 2/polycomb repressor complex 2 complex leads to sustained covalently closed circular DNA and host HCC‐related gene transcription [24]. However, reports on circulating *DLEU2* as a biomarker of HCC are limited. Here, we report its discriminant capacity highlighting its potential for use as a biomarker of very early‐stage HCC.

Increased expression of *HOTTIP* in HCC tissue compared to nontumor counterparts is reportedly associated with HCC progression and disease outcomes [25]. Additionally, *HOTTIP* may promote HCC carcinogenesis through targeting the miR‐125b [26], whereas miR‐192 and miR‐204 were suggested as upstream regulators for the suppression of *HOTTIP* expression in HCC [27]. Recently, a study showed significantly higher expression of *HOTTIP* in patients with advanced‐stage HCC, old age, male gender, white race, and no cirrhosis. Moreover, *HOTTIP* was expressed with genes related to the PPAR signaling pathway [28], which plays a critical role in the pathogenesis of HCC. Studies have also reported *HOTTIP* as a diagnostic or prognostic biomarker in gastric and colorectal cancers [29, 30]; however, to date, only one study has reported the discriminatory role of circulating *HOTTIP* in liver diseases. This study showed an increased expression of *HOTTIP* in resolved HBV patients compared to healthy controls [31].

*SNHG1* is also upregulated in HCC [32] and contributes to the development and progression of HCC via direct inhibition of miR‐195 expression [33]. *SNHG1* also has important roles in HCC cell cycle, migration, and epithelial–mesenchymal transition by epigenetic silencing of cyclin‐dependent kinase inhibitor (CKI) 1A and CKI2B [34]. Upregulated *SNHG1* reduces sorafenib‐induced apoptosis and autophagy in sorafenib‐resistant HCC cells by triggering the Akt pathway through the solute carrier family 3 member 2 [35]. It has also been reported that plasma *SNHG1* is elevated in HCC subjects and correlates with tissue *SNHG1* expression. The AUC was 0.86 (95% CI = 0.78–0.91) for HCC discrimination (HCC vs. CHB and LC) in combination with AFP [32].

Collectively, this is the first study to show the diagnostic role of circulating small EV‐derived *MALAT1*, *DLEU2*, *HOTTIP,* and *SNHG1*, which are reported onco‐lncRNAs in HCC. Interestingly, serum lncRNA levels did not differ significantly between subjects with HCC and those without, whereas serum EV‐derived lncRNA levels were significantly higher in subjects with HCC.

The proportion of EV‐encapsulated vs. free noncoding RNAs found in liquid biopsies is currently a controversial issue as researchers have shown that most microRNAs are integrated into ribonucleoprotein complexes, while only a small proportion are enclosed in EVs [36]. Meanwhile, others have demonstrated consistently higher concentrations of microRNAs in exosomes compared to that in free form within serum and saliva [37]. Our previous study also reported a higher level of small EV‐derived lncRNA than free serum lncRNA [12]. Therefore, both free and EV‐enclosed lncRNAs should be considered in an oncogenic liquid biopsy‐based biomarker study.

Certain limitations were noted in this study. First, the detected EV‐derived lncRNAs may have originated from cancer cells or from other cells, such as platelets or leukocytes. Indeed, the selective detection of tumor‐derived EVs from blood using specific surface markers prior to the analysis of lncRNAs has been a challenging issue in the research community. Second, the number of enrolled patients was relatively small, while the major etiology of HCC was limited to HBV (88.9%). Therefore, our results should be confirmed with a large number of external cohorts based on various etiologies.

## 5 Conclusions

Serum small EV‐derived *MALAT1*, *DLEU2*, *HOTTIP*, and *SNHG1* were significantly highly expressed in patients with HCC and showed good to excellent discriminating capacity that was significantly higher than that of the traditional tumor marker AFP. Furthermore, even in very early HCC with low AFP levels (≤ 20 ng·mL^−1^), EV‐derived *MALAT1*, *DLEU2*, *HOTTIP,* and *SNHG1* showed high positivity, suggesting the utility of EV‐lncRNAs as a diagnostic liquid biopsy biomarker for very early HCC, particularly in patients without AFP elevation.

## Conflict of interest

The authors declare no conflict of interest.

## Author contributions

JWE, SWN, and JYC conceived the study; JWE developed the methodology; GOB, HRA, and MKY acquired and analyzed the data; SSK, JWE, JAS, and GOB wrote the manuscript; HJC and JHY constructed the databases; and JYC supervised the study. All authors reviewed the manuscript and approved its submission for publication.

### Peer Review

The peer review history for this article is available at https://publons.com/publon/10.1002/1878‐0261.13049.

## Supporting information

**Fig. S1.** Communication between HCC and normal liver cells through EVs.
**Fig. S2.** Differential gene expression of final four serum EV‐lncRNAs.
**Fig. S3.** Age‐related EV‐derived lncRNA expression in the validation cohort in all patients.
**Fig. S4.** Prognostic power of four serum EV‐lncRNA expression in the validation cohort.
**Table S1.** Primer sequences in this study.
**Table S2.** AUROCs of combination of two markers for diagnosing HCC.

## Data Availability

The data used or analyzed in this article are available from the corresponding author upon reasonable request.

## References

[1] Bray F, Ferlay J, Soerjomataram I, Siegel RL, Torre LA & Jemal A (2018) Global cancer statistics 2018: GLOBOCAN estimates of incidence and mortality worldwide for 36 cancers in 185 countries. CA Cancer J Clin 68, 394–424. 30207593 10.3322/caac.21492

[2] Petrick JL & McGlynn KA (2019) The changing epidemiology of primary liver cancer. Curr Epidemiol Rep 6, 104–111. 31259140 10.1007/s40471-019-00188-3 PMC6599615

[3] Schwabe RF & Wang TC (2011) Targeting liver cancer: first steps toward a miracle? Cancer Cell 20, 698–699. 22172719 10.1016/j.ccr.2011.11.021 PMC3619204

[4] Tzartzeva K, Obi J, Rich NE, Parikh ND, Marrero JA, Yopp A, Waljee AK & Singal AG (2018) Surveillance imaging and alpha fetoprotein for early detection of hepatocellular carcinoma in patients with cirrhosis: a meta‐analysis. Gastroenterology 154, 1706–1718, e1701. 29425931 10.1053/j.gastro.2018.01.064 PMC5927818

[5] Becker A, Thakur BK, Weiss JM, Kim HS, Peinado H & Lyden D (2016) Extracellular vesicles in cancer: cell‐to‐cell mediators of metastasis. Cancer Cell 30, 836–848. 27960084 10.1016/j.ccell.2016.10.009 PMC5157696

[6] O'Driscoll L (2015) Expanding on exosomes and ectosomes in cancer. N Engl J Med 372, 2359–2362. 26061842 10.1056/NEJMcibr1503100

[7] Dahariya S, Paddibhatla I, Kumar S, Raghuwanshi S, Pallepati A & Gutti RK (2019) Long non‐coding RNA: classification, biogenesis and functions in blood cells. Mol Immunol 112, 82–92. 31079005 10.1016/j.molimm.2019.04.011

[8] Quinn JJ & Chang HY (2016) Unique features of long non‐coding RNA biogenesis and function. Nat Rev Genet 17, 47–62. 26666209 10.1038/nrg.2015.10

[9] Gutschner T & Diederichs S (2012) The hallmarks of cancer: a long non‐coding RNA point of view. RNA Biol 9, 703–719. 22664915 10.4161/rna.20481 PMC3495743

[10] Mercer TR, Dinger ME & Mattick JS (2009) Long non‐coding RNAs: Insights into functions. Nat Rev Genet 10, 155–159. 19188922 10.1038/nrg2521

[11] Marrero JA, Kulik LM, Sirlin CB, Zhu AX, Finn RS, Abecassis MM, Roberts LR & Heimbach JK (2018) Diagnosis, staging, and management of hepatocellular carcinoma: 2018 practice guidance by the American Association for the Study of Liver Diseases. Hepatology 68, 723–750. 29624699 10.1002/hep.29913

[12] Kim SS, Baek GO, Ahn HR, Sung S, Seo CW, Cho HJ, Nam SW, Cheong JY & Eun JW (2020) Serum small extracellular vesicle‐derived LINC00853 as a novel diagnostic marker for early hepatocellular carcinoma. Mol Oncol 14, 2646–2659. 32525601 10.1002/1878-0261.12745 PMC7530776

[13] Mei H, Liu Y, Zhou Q, Hu K & Liu Y (2019) Long noncoding RNA MALAT1 acts as a potential biomarker in cancer diagnosis and detection: a meta‐analysis. Biomark Med 13, 45–54. 30561226 10.2217/bmm-2018-0128

[14] Tripathi V, Ellis JD, Shen Z, Song DY, Pan Q, Watt AT, Freier SM, Bennett CF, Sharma A, Bubulya PA*et al*. (2010) The nuclear‐retained noncoding RNA MALAT1 regulates alternative splicing by modulating SR splicing factor phosphorylation. Mol Cell 39, 925–938. 20797886 10.1016/j.molcel.2010.08.011 PMC4158944

[15] Wang J, Su L, Chen X, Li P, Cai Q, Yu B, Liu B, Wu W & Zhu Z (2014) MALAT1 promotes cell proliferation in gastric cancer by recruiting SF2/ASF. Biomed Pharmacother 68, 557–564. 24857172 10.1016/j.biopha.2014.04.007

[16] West JA, Davis CP, Sunwoo H, Simon MD, Sadreyev RI, Wang PI, Tolstorukov MY & Kingston RE (2014) The long noncoding RNAs NEAT1 and MALAT1 bind active chromatin sites. Mol Cell 55, 791–802. 25155612 10.1016/j.molcel.2014.07.012 PMC4428586

[17] Hou Z, Xu X, Fu X, Tao S, Zhou J, Liu S & Tan D (2017) Hbx‐related long non‐coding RNA MALAT1 promotes cell metastasis via up‐regulating LTBP3 in hepatocellular carcinoma. Am J Cancer Res 7, 845–856. 28469957 PMC5411792

[18] Li C, Miao R, Liu S, Wan Y, Zhang S, Deng Y, Bi J, Qu K, Zhang J & Liu C (2017) Down‐regulation of mir‐146b‐5p by long noncoding RNA MALAT1 in hepatocellular carcinoma promotes cancer growth and metastasis. Oncotarget 8, 28683–28695. 28404923 10.18632/oncotarget.15640 PMC5438683

[19] Liu D, Zhu Y, Pang J, Weng X, Feng X & Guo Y (2018) Knockdown of long non‐coding RNA MALAT1 inhibits growth and motility of human hepatoma cells via modulation of miR‐195. J Cell Biochem 119, 1368–1380. 28722813 10.1002/jcb.26297

[20] Huang J, Zheng Y, Xiao X, Liu C, Lin J, Zheng S, Yang B & Ou Q (2020) A circulating long noncoding RNA panel serves as a diagnostic marker for hepatocellular carcinoma. Dis Markers 2020, 5417598. 32733618 10.1155/2020/5417598 PMC7376401

[21] Konishi H, Ichikawa D, Yamamoto Y, Arita T, Shoda K, Hiramoto H, Hamada J, Itoh H, Fujita Y, Komatsu S*et al*. (2016) Plasma level of metastasis‐associated lung adenocarcinoma transcript 1 is associated with liver damage and predicts development of hepatocellular carcinoma. Cancer Sci 107, 149–154. 26614531 10.1111/cas.12854 PMC4768388

[22] Yuan W, Sun Y, Liu L, Zhou B, Wang S & Gu D (2017) Circulating lncRNAs serve as diagnostic markers for hepatocellular carcinoma. Cell Physiol Biochem 44, 125–132. 29130980 10.1159/000484589

[23] Guo Y, Bai M, Lin L, Huang J, An Y, Liang L, Liu Y & Huang W (2019) LncRNA DLEU2 aggravates the progression of hepatocellular carcinoma through binding to EZH2. Biomed Pharmacother 118, 109272. 31376657 10.1016/j.biopha.2019.109272

[24] Salerno D, Chiodo L, Alfano V, Floriot O, Cottone G, Paturel A, Pallocca M, Plissonnier M‐L, Jeddari S, Belloni L*et al*. (2020) Hepatitis b protein HBx binds the DLEU2 lncRNA to sustain cccDNA and host cancer‐related gene transcription. Gut 69, 2016–2024. 32114505 10.1136/gutjnl-2019-319637 PMC7569396

[25] Quagliata L, Matter MS, Piscuoglio S, Arabi L, Ruiz C, Procino A, Kovac M, Moretti F, Makowska Z, Boldanova T*et al*. (2014) Long noncoding RNA HOTTIP/HOXA13 expression is associated with disease progression and predicts outcome in hepatocellular carcinoma patients. Hepatology 59, 911–923. 24114970 10.1002/hep.26740 PMC3943759

[26] Tsang FH, Au SL, Wei L, Fan DN, Lee JM, Wong CC, Ng IO & Wong CM (2015) Long non‐coding RNA HOTTIP is frequently up‐regulated in hepatocellular carcinoma and is targeted by tumour suppressive miR‐125b. Liver Int 35, 1597–1606. 25424744 10.1111/liv.12746

[27] Ge Y, Yan X, Jin Y, Yang X, Yu X, Zhou L, Han S, Yuan Q & Yang M (2015) MiRNA‐192 [corrected] and MiRNA‐204 directly suppress lncRNA Hottip and interrupt GLS1‐mediated glutaminolysis in hepatocellular carcinoma. PLoS Genet 11, e1005726. 26710269 10.1371/journal.pgen.1005726 PMC4692503

[28] Zhang Y, Huang JC, Cai KT, Yu XB, Chen YR, Pan WY, He ZL, Lv J, Feng ZB & Chen G (2017) Long non‐coding RNA HOTTIP promotes hepatocellular carcinoma tumorigenesis and development: a comprehensive investigation based on bioinformatics, qRT‐PCR and meta‐analysis of 393 cases. Int J Oncol 51, 1705–1721. 29039502 10.3892/ijo.2017.4164 PMC5673011

[29] Oehme F, Krahl S, Gyorffy B, Muessle B, Rao V, Greif H, Ziegler N, Lin K, Thepkaysone ML, Polster H*et al*. (2019) Low level of exosomal long non‐coding RNA HOTTIP is a prognostic biomarker in colorectal cancer. RNA Biol 16, 1339–1345. 31251124 10.1080/15476286.2019.1637697 PMC6779381

[30] Zhao R, Zhang Y, Zhang X, Yang Y, Zheng X, Li X, Liu Y & Zhang Y (2018) Exosomal long noncoding RNA HOTTIP as potential novel diagnostic and prognostic biomarker test for gastric cancer. Mol Cancer 17, 68. 29486794 10.1186/s12943-018-0817-x PMC6389063

[31] Yilmaz Susluer S, Kayabasi C, Ozmen Yelken B, Asik A, Celik D, Balci Okcanoglu T, Serin Senger S, Biray Avci C, Kose S & Gunduz C (2018) Analysis of long non‐coding RNA (lncRNA) expression in hepatitis B patients. Bosn J Basic Med Sci 18, 150–161. 29669510 10.17305/bjbms.2018.2800 PMC5988534

[32] Gao S, Xu X, Wang Y, Zhang W & Wang X (2018) Diagnostic utility of plasma lncRNA small nucleolar RNA host gene 1 in patients with hepatocellular carcinoma. Mol Med Rep 18, 3305–3313. 30066898 10.3892/mmr.2018.9336 PMC6102699

[33] Zhang H, Zhou D, Ying M, Chen M, Chen P, Chen Z & Zhang F (2016) Expression of long non‐coding RNA (lncRNA) small nucleolar RNA host gene 1 (SNHG1) exacerbates hepatocellular carcinoma through suppressing miR‐195. Med Sci Monit 22, 4820–4829. 27932778 10.12659/MSM.898574 PMC5167104

[34] Li B, Li A, You Z, Xu J & Zhu S (2020) Epigenetic silencing of CDKN1A and CDKN2B by SNHG1 promotes the cell cycle, migration and epithelial‐mesenchymal transition progression of hepatocellular carcinoma. Cell Death Dis 11, 823. 33009370 10.1038/s41419-020-03031-6 PMC7532449

[35] Li W, Dong X, He C, Tan G, Li Z, Zhai BO, Feng J, Jiang X, Liu C, Jiang H*et al*. (2019) LncRNA SNHG1 contributes to sorafenib resistance by activating the Akt pathway and is positively regulated by miR‐21 in hepatocellular carcinoma cells. J Exp Clin Cancer Res 38, 183. 31053148 10.1186/s13046-019-1177-0 PMC6499991

[36] Arroyo JD, Chevillet JR, Kroh EM, Ruf IK, Pritchard CC, Gibson DF, Mitchell PS, Bennett CF, Pogosova‐Agadjanyan EL, Stirewalt DL*et al*. (2011) Argonaute2 complexes carry a population of circulating micrornas independent of vesicles in human plasma. Proc Natl Acad Sci USA 108, 5003–5008. 21383194 10.1073/pnas.1019055108 PMC3064324

[37] Gallo A, Tandon M, Alevizos I & Illei GG (2012) The majority of microRNAs detectable in serum and saliva is concentrated in exosomes. PLoS One 7, e30679. 22427800 10.1371/journal.pone.0030679 PMC3302865

