# Supplementary material for: Early detection of hepatocellular carcinoma via liquid biopsy: panel of small extracellular vesicle‐derived long noncoding RNAs identified as markers
Source: Mol Oncol. 2021 Jul 12;15(10):2715–31. doi: 10.1002/1878-0261.13049 (PMC8486572; doi:10.1002/1878-0261.13049)
Supplement: Supplementary file 1 — Fig. S1. Communication between HCC and normal liver cells through EVs. Fig. S2. Differential gene expression of final four serum EV‐lncRNAs. Fig. S3. Age‐related EV‐derived lncRNA expression in the validation cohort in all patients. Fig. S4. Prognostic power of four serum EV‐lncRNA expression in the validation cohort. Table S1. Primer sequences in this study. Table S2. AUROCs of combination of two markers for diagnosing HCC. [file MOL2-15-2715-s001.docx]

**Supporting Table 1. Primer sequences in this study**

| Gene | Accession No. | Forward sequence | Reverse sequence |
| --- | --- | --- | --- |
| *DLEU2* | NR_152566.1 | 5’-TCTGGAGAACAGCCTCACTTC-3’ | 5’-TGCTGAGCTAAGTAGAGGTCTC-3’ |
| *HOTTIP* | NR_037843.3 | 5’-CAGGAACCGGCGCGTATTTC-3’ | 5’-CCCAGGCATATCCGCAGGAA-3’ |
| *MALAT1* | NR_002819.4 | 5’-GAATTGCGTCATTTAAAGCCTAGTT-3’ | 5’-GTTTCATCCTACCACTCCCAATTAAT-3’ |
| *NEAT1(β)* | NR_131012.1 | 5’-GTTGGCAGCACTGAGAACCG-3’ | 5’-AGCCCTGGCATTAAGGGTCC-3’ |
| *SNHG1* | NR_003098.2 | 5’-CAGGCCCAGAACCCAAACTC-3’ | 5’-GGCTCATGACGGGAACAGAA-3’ |
| *TUG1* | NR_110492.1 | 5’-CCTGGACCTGGAACCCGAAA-3’ | 5’-TGCTGGTGGTAGTGCTTGCT-3’ |
| *HMBS* | NM_001024382.2 | 5’-GGAGGGCAGAAGGAAGAAAACAG-3’ | 5’-CACTGTCCGTCTGTATGCGAG-3’ |

**Supporting Table 2. AUROCs of combination of two markers for diagnosing HCC**

| Non tumor (n=67) vs HCC (n=72) | | | |  | CH/LC (n=46) vs HCC (n=72) | | | |
| --- | --- | --- | --- | --- | --- | --- | --- | --- |
|  | AUC | SE | 95% CI |  |  | AUC | SE | 95% CI |
| AFP | 0.647 | 0.048 | 0.553 to 0.741 |  | AFP | 0.506 | 0.0563 | 0.395 to 0.616 |
| AFP+DLEU2 | 0.89 | 0.0264 | 0.838 to 0.941 |  | AFP+DLEU2 | 0.851 | 0.0346 | 0.783 to 0.919 |
| AFP+HOTTIP | 0.882 | 0.028 | 0.827 to 0.937 |  | AFP+HOTTIP | 0.845 | 0.0348 | 0.777 to 0.913 |
| **AFP+MALAT1** | **0.911** | **0.0238** | **0.864 to 0.958** |  | AFP+MALAT1 | 0.886 | 0.0297 | 0.828 to 0.945 |
| AFP+SNHG1 | 0.899 | 0.0249 | 0.851 to 0.948 |  | AFP+SNHG1 | 0.867 | 0.0325 | 0.804 to 0.931 |
| DLEU2+HOTTIP | 0.888 | 0.0262 | 0.837 to 0.940 |  | DLEU2+HOTTIP | 0.85 | 0.0341 | 0.783 to 0.917 |
| DLEU2+MALAT1 | 0.909 | 0.0245 | 0.861 to 0.957 |  | DLEU2+MALAT1 | 0.886 | 0.0298 | 0.828 to 0.945 |
| DLEU2+SNHG1 | 0.893 | 0.0261 | 0.842 to 0.944 |  | DLEU2+SNHG1 | 0.859 | 0.034 | 0.792 to 0.925 |
| HOTTIP+MALAT1 | 0.908 | 0.0244 | 0.860 to 0.956 |  | HOTTIP+MALAT1 | 0.884 | 0.03 | 0.826 to 0.943 |
| HOTTIP+SNHG1 | 0.898 | 0.0249 | 0.849 to 0.947 |  | HOTTIP+SNHG1 | 0.864 | 0.0327 | 0.800 to 0.928 |
| MALAT1+SNHG1 | 0.909 | 0.0245 | 0.861 to 0.957 |  | **MALAT1+SNHG1** | **0.887** | **0.0298** | **0.828 to 0.945** |
|  |  |  |  |  |  |  |  |  |
| Non tumor (n=67) vs mUICC I/II (n=37) | | | |  | CH/LC (n=46) vs mUICC I/II (n=37) | | | |
|  | AUC | SE | 95% CI |  |  | AUC | SE | 95% CI |
| AFP | 0.531 | 0.0575 | 0.418 to 0.643 |  | AFP | 0.351 | 0.0626 | 0.229 to 0.473 |
| AFP+DLEU2 | 0.889 | 0.032 | 0.826 to 0.951 |  | AFP+DLEU2 | 0.851 | 0.0421 | 0.768 to 0.933 |
| AFP+HOTTIP | 0.873 | 0.0366 | 0.801 to 0.945 |  | AFP+HOTTIP | 0.832 | 0.0449 | 0.744 to 0.920 |
| AFP+MALAT1 | 0.916 | 0.03 | 0.857 to 0.975 |  | AFP+MALAT1 | 0.891 | 0.0378 | 0.817 to 0.965 |
| AFP+SNHG1 | 0.897 | 0.0303 | 0.838 to 0.956 |  | AFP+SNHG1 | 0.864 | 0.0395 | 0.787 to 0.941 |
| DLEU2+HOTTIP | 0.899 | 0.0299 | 0.840 to 0.957 |  | DLEU2+HOTTIP | 0.862 | 0.0395 | 0.784 to 0.939 |
| **DLEU2+MALAT1** | **0.919** | **0.0294** | **0.861 to 0.976** |  | **DLEU2+MALAT1** | **0.895** | **0.0369** | **0.823 to 0.968** |
| DLEU2+SNHG1 | 0.905 | 0.0295 | 0.847 to 0.963 |  | DLEU2+SNHG1 | 0.871 | 0.0389 | 0.795 to 0.948 |
| HOTTIP+MALAT1 | 0.92 | 0.0288 | 0.863 to 0.976 |  | **HOTTIP+MALAT1** | **0.895** | **0.0367** | **0.823 to 0.967** |
| HOTTIP+SNHG1 | 0.906 | 0.0273 | 0.853 to 0.960 |  | HOTTIP+SNHG1 | 0.874 | 0.0364 | 0.802 to 0.945 |
| MALAT1+SNHG1 | 0.918 | 0.0295 | 0.860 to 0.976 |  | **MALAT1+SNHG1** | **0.895** | **0.0371** | **0.823 to 0.968** |
|  |  |  |  |  |  |  |  |  |
| Non tumor (n=67) vs mUICC I (n=28) | | | |  | CH/LC (n=46) vs mUICC I (n=28) | | | |
|  | AUC | SE | 95% CI |  |  | AUC | SE | 95% CI |
| AFP | 0.508 | 0.0602 | 0.390 to 0.626 |  | AFP | 0.328 | 0.0636 | 0.205 to 0.452 |
| AFP+DLEU2 | 0.879 | 0.037 | 0.806 to 0.952 |  | AFP+DLEU2 | 0.838 | 0.0485 | 0.743 to 0.933 |
| AFP+HOTTIP | 0.872 | 0.0425 | 0.789 to 0.955 |  | AFP+HOTTIP | 0.832 | 0.0507 | 0.733 to 0.932 |
| AFP+MALAT1 | 0.919 | 0.0341 | 0.852 to 0.986 |  | AFP+MALAT1 | 0.895 | 0.0427 | 0.811 to 0.979 |
| AFP+SNHG1 | 0.901 | 0.0322 | 0.838 to 0.964 |  | AFP+SNHG1 | 0.869 | 0.042 | 0.787 to 0.951 |
| DLEU2+HOTTIP | 0.891 | 0.0344 | 0.824 to 0.959 |  | DLEU2+HOTTIP | 0.852 | 0.0454 | 0.763 to 0.941 |
| **DLEU2+MALAT1** | **0.921** | **0.0338** | **0.854 to 0.987** |  | DLEU2+MALAT1 | 0.898 | 0.0422 | 0.816 to 0.981 |
| DLEU2+SNHG1 | 0.899 | 0.0341 | 0.832 to 0.966 |  | DLEU2+SNHG1 | 0.863 | 0.0448 | 0.775 to 0.950 |
| **HOTTIP+MALAT1** | **0.921** | **0.033** | **0.856 to 0.986** |  | HOTTIP+MALAT1 | 0.896 | 0.042 | 0.814 to 0.978 |
| HOTTIP+SNHG1 | 0.906 | 0.0296 | 0.848 to 0.964 |  | HOTTIP+SNHG1 | 0.873 | 0.0394 | 0.795 to 0.950 |
| MALAT1+SNHG1 | 0.92 | 0.0341 | 0.853 to 0.987 |  | **MALAT1+SNHG1** | **0.899** | **0.0424** | **0.816 to 0.982** |


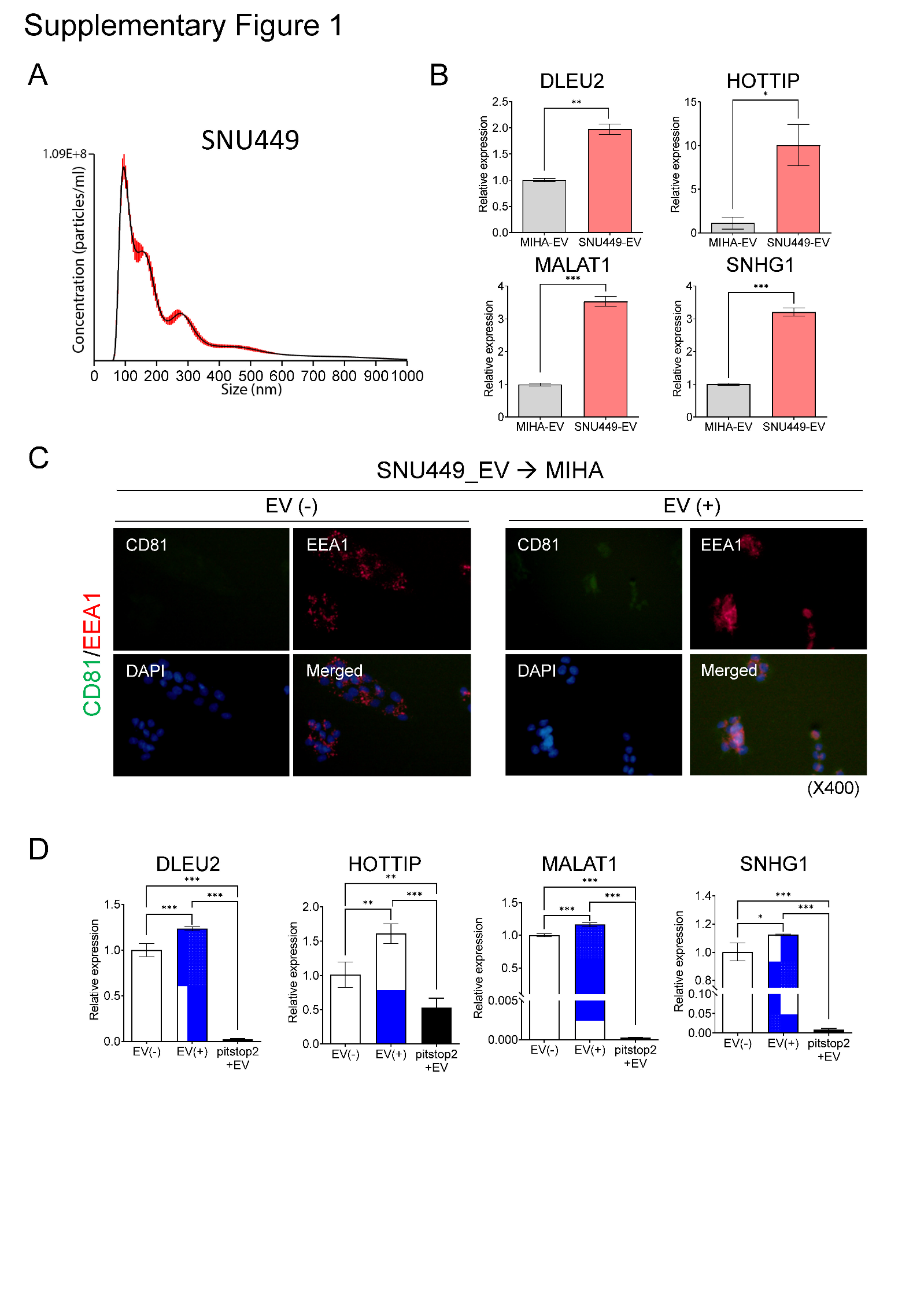


Figure S1. Communication between HCC and normal liver cells through EVs. (A) NTA result of SNU449-derived EV. (B) Four lncRNAs expression of MIHA-EV and SNU449-EV. (C) After delivery of SNU449-EV to MIHA, microscopy images showed overlapping of green (CD8; EV marker), red (EEA1; endosome marker) and blue (DAPI) fluorescence. (F) Four EV-lncRNAs expression analysis by qRT‐PCR. All lncRNAs expression levels were significantly increased in EV-treated MIHA, and decreased after endocytosis inhibitor (pitstop 2) treatment.


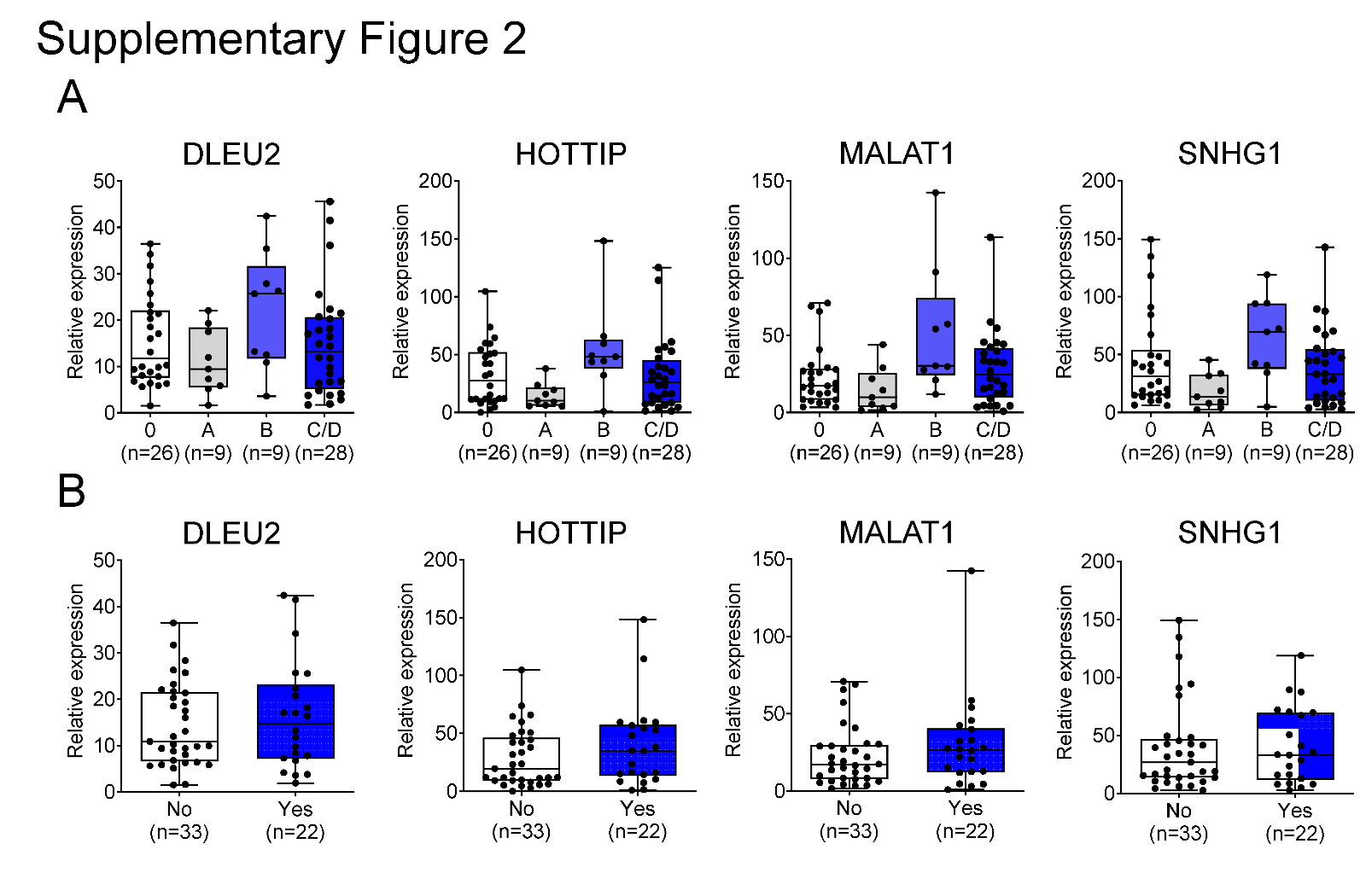
Figure S2. Differential gene expression of final four serum EV-lncRNAs. (A) Box plot showing the expression of four serum EV-lncRNAs according to BCLC stage in the validation cohort (n=70) (B) Box plot showing the expression of four serum EV-lncRNAs according to vascular invasion status in the validation cohort (n=55).


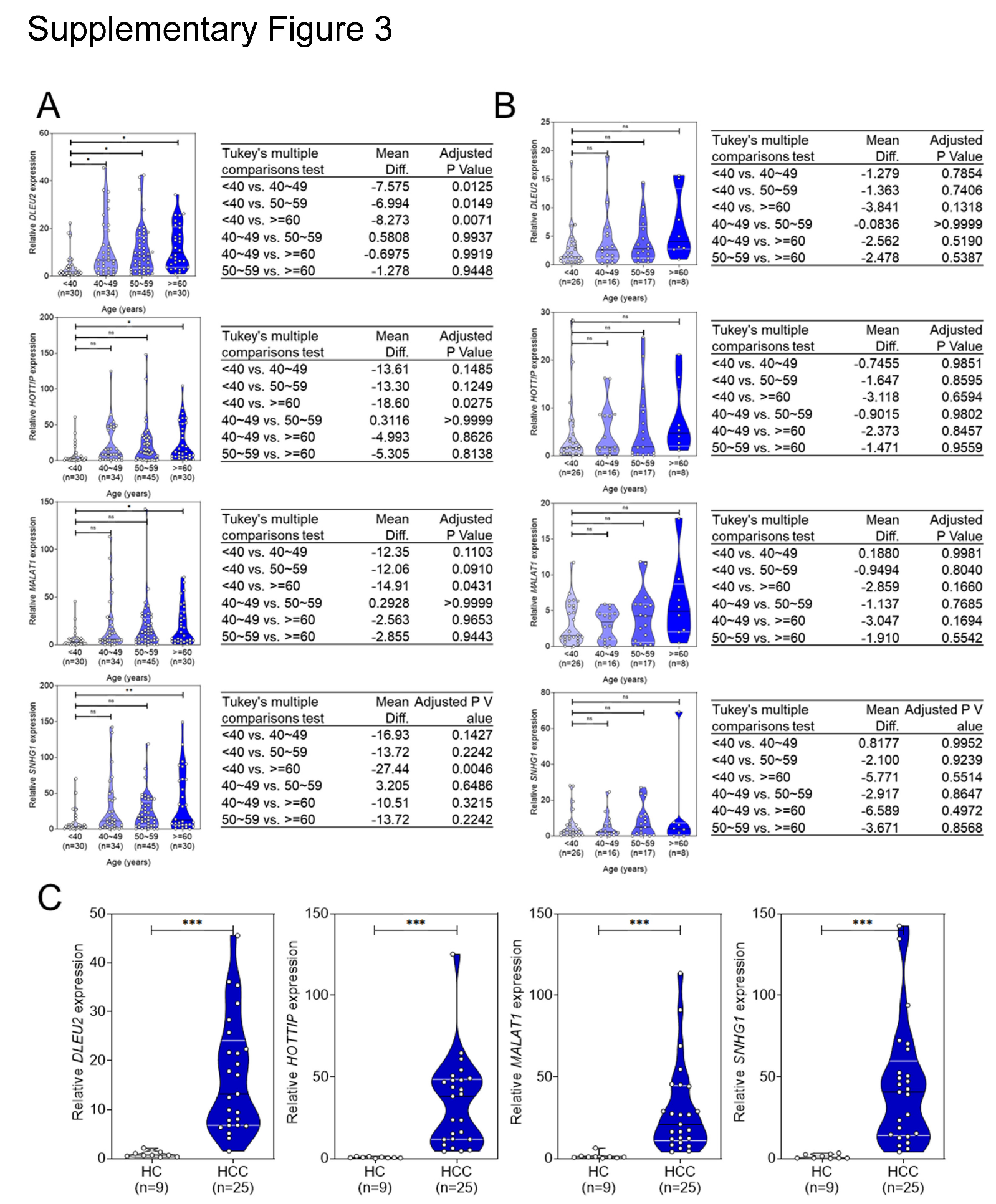


Figure S3. Age-related EV-derived lncRNAs expression in the validation cohort in all patients (A) and patients without HCC (B). Expression of EV-lncRNAs expression in healthy control (HC) and patients with HCC with same age distribution (33-51 years).


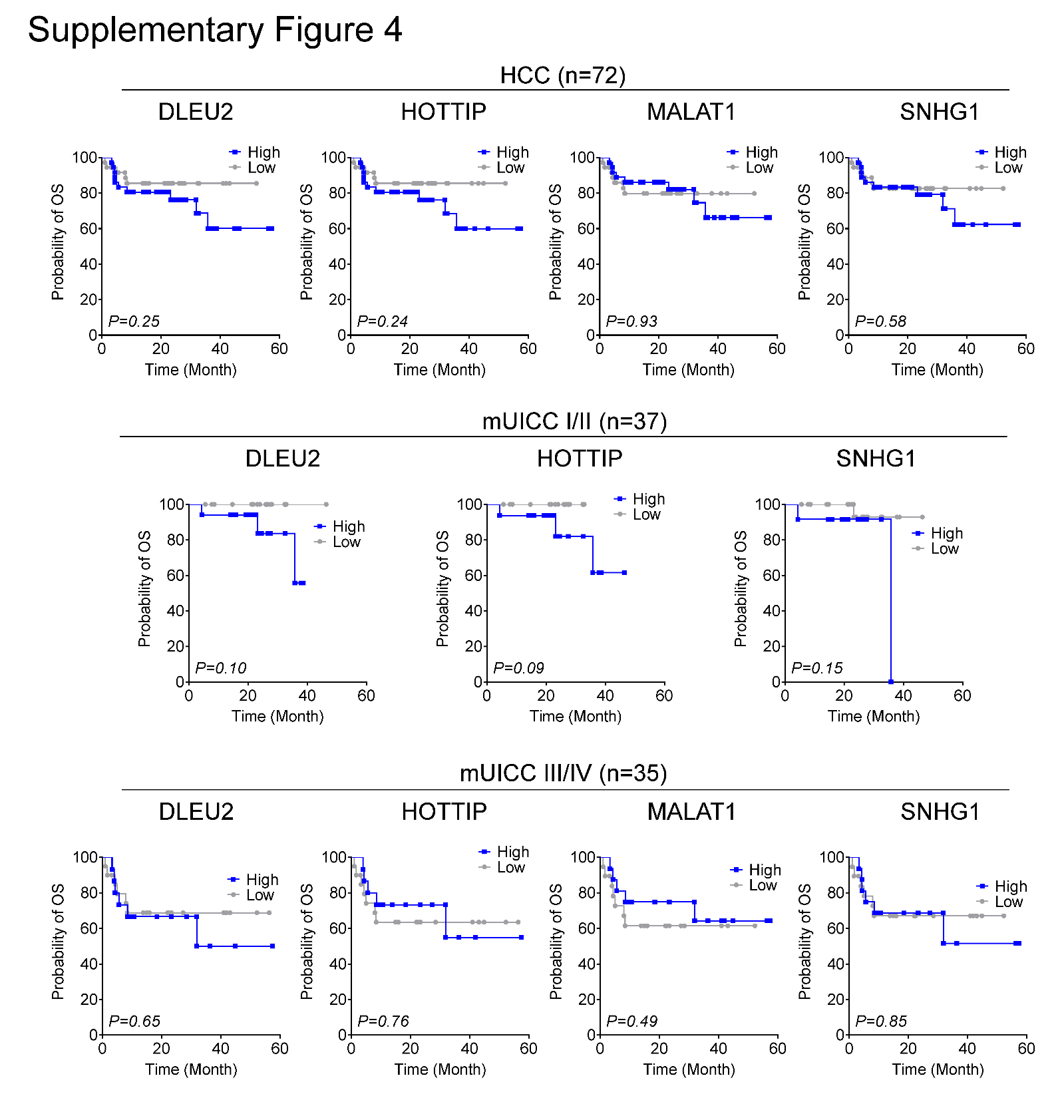


Figure S4. Prognostic power of four serum EV-lncRNAs expression in the validation cohort.
